# Joint meta-analysis of two diagnostic tests accounting for within and between studies dependence

**DOI:** 10.1177/09622802241269645

**Published:** 2024-09-11

**Authors:** Aristidis K Nikoloulopoulos

**Affiliations:** 6106 Department of Mathematics, School of Engineering, Mathematics and Physics, University of East Anglia, Norwich, UK

**Keywords:** Mixed models, multinomial generalised linear mixed model, multiple diagnostic tests, multivariate meta-analysis, summary receiver operating characteristic curves, vine copula models

## Abstract

There is an extensive literature on methods for meta-analysis of diagnostic test accuracy, but it mainly focuses on a single test. A multinomial generalised linear mixed model was recently proposed for the joint meta-analysis of studies comparing two tests on the same participants in a paired tests design with a gold standard. In this setting, we propose a novel model for joint meta-analysis of studies comparing two diagnostic tests which assumes independent multinomial distributions for the counts of each combination of test results in diseased and non-diseased patients, conditional on the latent vector of probabilities of each combination of test results in diseased and non-diseased patients. For the random effects distribution of the latent proportions, we employ a one-truncated D-vine copula that can provide tail dependence or asymmetry. The proposed model includes the multinomial generalised linear mixed model as a special case, accounts for the within-study dependence induced because the tests are applied to the same participants, allows for between-studies dependence, and can also operate on the original scale of the latent proportions. The latter enables the derivation of summary receiver operating characteristic curves. Our methodology is demonstrated with simulation studies and a meta-analysis of screening for Down’s syndrome with two tests: shortened humerus and shortened femur.

## 1. Introduction

Diagnostic test accuracy studies aim to quantify the diagnostic accuracy of a new test in relation to the current perfect reference standard, also known as the gold standard. The development of an accurate diagnostic test can lead to early detection of a specific disease, which can reduce healthcare costs in the long term. For example, if a diagnostic test can detect cancer at an early stage, before it has spread to other parts of the body, the chances of successful treatment are much higher. This can reduce the need for more expensive and invasive treatments such as surgery, radiation therapy, or chemotherapy, which can be associated with high healthcare costs.^1^ Furthermore, early detection can also help to prevent the spread of infectious diseases such as COVID-19, which can have a significant impact on healthcare costs. By identifying infected individuals early, healthcare providers can implement appropriate isolation measures to prevent the spread of the disease to others.^2^

The large number of available diagnostic test accuracy studies has led to the use of meta-analysis as an integrated analysis to detect an accurate diagnostic test versus an analysis based on a single study. As the accuracy of a diagnostic test is commonly measured by a pair of indices such as the true positive fraction (TPF, the probability that an actual positive will test positive) and false positive fraction (FPF, the complementary probability that an actual negative will test negative), synthesis of diagnostic test accuracy studies requires multivariate meta-analysis methods.^3^ There is an extensive literature on methods for meta-analysis of diagnostic studies. All studies evaluate the accuracy of the same diagnostic test when a gold standard is available, but they mainly focus on a single test.^4–8^ However, the better understanding of a particular disease, along with the technological advances in many health sectors has led to the development of multiple tests. Meta-analysis of multiple diagnostic tests can help to identify the most accurate diagnostic test or combination of tests for a specific condition or disease. This can help clinicians to make more informed decisions about which tests to use, which can improve patient outcomes and reduce healthcare costs.^9^ For example, a meta-analysis of studies evaluating the accuracy of different tests for diagnosing COVID-19 can help healthcare providers to determine which tests are most reliable and accurate, and which tests should be prioritised in different settings or patient populations. This can have a significant impact on patient health by ensuring that accurate diagnoses are made and appropriate treatments are provided in a timely manner.^10^

As summarised by Takwoingi et al.,^11^ diagnostic test accuracy studies are comparative when they assess two or more tests. The robust comparative studies of diagnostic test accuracy use either a paired test (also called multiple or crossover) design, in which all patients undergo all tests together with the perfect reference standard, or more rarely, a randomised (also called parallel) design, in which all patients undergo the perfect reference standard test but are randomly allocated to have only one of the other tests. In this paper, we deal with the joint meta-analysis of studies comparing two diagnostic tests in a paired test design. We consider the case where the numbers of all different combinations of the test results are given, that is, there are four possible combinations of positive and negative results with different frequencies for individuals with or without the target condition. These frequencies are denoted by $y_{ijkd},\;i=1,\ldots ,N,\;j=0,1,\;k=0,1,\;d=0,1$ , where i is an index for the individual studies, j is an index for the test 1 outcome (0: negative; 1: positive), k is an index for the test 2 outcome (0: negative; 1: positive) and d is an index for the disease status (0: non-diseased; 1: diseased). The “classic” 2×2 table showing the cross-classification of the reference standard result and the index test result is extended to a 4×2 table (Table 1) that cross-classifies the results of two index tests being compared within diseased and non-diseased participants. Each cell in Table 1 provides the cell frequency corresponding to a combination of index tests and disease outcome in study i . The additional modelling of the information between the two tests strategically allows to account for potential within-study dependence that can occur because every study participant underwent both diagnostic tests. This is an important feature that other proposed models for meta-analysis and comparison of two diagnostic tests fail to fulfil as they are solely based on two 2×2 tables per study, that is, they do not consider cross-classified test results.^12,13^

**Table 1.** Data from an individual study in a 4×2 table.

		Disease (by gold standard)	
Test 1	Test 2	+	−
+	−	$y_{i101}$	$y_{i100}$
−	+	$y_{i011}$	$y_{i010}$
+	+	$y_{i111}$	$y_{i110}$
−	−	$y_{i001}$	$y_{i000}$
Total		$y_{i++1}$	$y_{i++0}$

Trikalinos et al.^14^ proposed a multinomial generalised linear mixed model (GLMM) for the joint meta-analysis of two tests. Their model assumes independent multinomial distributions for the counts of each combination of test results in diseased patients and the counts of each combination of test results in non-diseased patients in Table 1, conditional on the six-variate normally distributed transformed latent TPF and FPF for each test, and latent joint TPF and FPF, which capture information on the agreement between the two tests in each study.

Nevertheless, the six-variate normal distribution of the transformed latent proportions in the multinomial GLMM has restricted properties, that is, a linear correlation structure and normal margins that might lead to biased meta-analytic estimates of diagnostic test accuracy. In order to create a flexible distribution to model the random effects we exploit the use of regular vine copulas,^15^ as other parametric copulas such as Archimedean, nested Archimedean and elliptical copulas have limited dependence.^16^ Regular vine copulas are suitable for high-dimensional data,^17^ hence given the low dimension, we use their boundary case, namely a D-vine copula. D-vine copulas have become important in many applications areas such as finance^18,19^ and biological sciences,^20,21^ to name just a few, in order to deal with dependence in the joint tails. Another boundary case of regular vine copulas is the canonical vine copula, but this parametric family of copulas is suitable if there exists a pilot variable that drives the dependence among the variables,^22–24^ which apparently is not the case in this application area as none of the aforementioned variables in Table 1 is a pilot variable.

We propose a multinomial copula mixed model (CMM) as an extension of the multinomial GLMM by using a D-vine copula representation of the random effects distribution with normal and beta margins. We assume independent multinomial distributions for the counts of each combination of test results in diseased patients and the counts of each combination of test results in non-diseased patients, conditional on the latent probabilities of each combination of test results in diseased and non-diseased patients in each study. We consider the case where the same individuals receive both tests, and the results are cross-classified. The proposed model (a) includes the multinomial GLMM^14^ as a special case, (b) accounts for the within-study dependence induced because the tests are applied to the same participants, (c) can have arbitrary univariate distributions for the random effects, and (d) can provide between-studies tail dependencies and asymmetries. The proposed model extends the model by Nikoloulopoulos^25^ for the meta-analysis of one diagnostic test with non-evaluable outcomes to six rather than four dimensions and to more than one test.

The remainder of the article proceeds as follows. Section 2 introduces the multinomial D-vine CMM for meta-analysis and comparison of two diagnostic tests, and provides computational details for maximum likelihood (ML) estimation. Section 3 studies the small-sample efficiency of the proposed ML estimation technique and investigates the effect of misspecifying the random effects distribution on parameter estimates and standard errors. Section 4 deduces summary receiver operating characteristic (SROC) curves from the proposed model through quantile regression techniques. Section 5 demonstrates our methodology by insightfully re-analysing the data from the systematic review that examined the screening accuracy of two second-trimester ultrasonographic tests that screen for Down’s syndrome. We conclude with some discussion in Section 6, followed by a brief section with software details.

## 2. The multinomial one-truncated D-vine CMM

In this section, we introduce the multinomial one-truncated D-vine CMM for the joint meta-analysis of two diagnostic tests and discuss its relationship with the multinomial GLMM. We complete this section with details on ML estimation.

### 2.1. D-vine copula representation of the random effects distribution

We assume that the counts $Y_{d}=(Y_{i10d},Y_{i01d},Y_{i11d},Y_{i00d})$ of each combination of test results in diseased ( d=1 ) or non-diseased ( d=0 ) patients are multinomially distributed given $X_{d}=x_{d}$ , where $X_{d}=(X_{10d},X_{01d},X_{11d})$ is the latent vector of transformed probabilities of each combination of test results in diseased ( d=1 ) or non-diseased ( d=0 ) patients. The counts $\left(Y_{i10d},Y_{i01d},Y_{i11d},Y_{i00d}\right)$ of each combination of test results in diseased ( d=1 ) or non-diseased ( d=0 ) patients are mutually exclusive outcomes. Since the four outcomes in each population are mutually exclusive, and one must occur, we have that their probabilities sum to one and hence we have three transformed probabilities $\left(X_{10d},X_{01d},X_{11d}\right)$ in diseased ( d=1 ) or non-diseased ( d=0 ) patients as the fourth can be derived by the other three.

**Figure 1.**
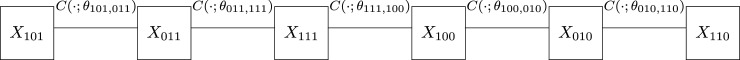
Graphical representation of the six-dimensional one-truncated D-vine copula model.

For the between-studies model, there are different latent variables $\left(X_{10d},X_{01d},X_{11d}\right)$ , but they are dependent. Hence the observed data $y_{ijkd}$ are dependent. In multivariate models with copulas, a copula or multivariate uniform distribution is combined with a set of univariate margins.^26^ This is equivalent to assuming that the latent variables $\left(X_{10d},X_{01d},X_{11d}\right)$ have been transformed to standard uniform latent variables $\left(U_{10d},U_{01d},U_{11d}\right)$ . So we assume that $\left(U_{101},U_{011},U_{111},U_{100},U_{010},U_{110}\right)$ is a six-dimensional random vector where $U_{10d},U_{01d},U_{11d}∼U(0,1)$ . The joint cdf is then given by $C_{6}(u_{101},u_{011},u_{111},u_{100},u_{010},u_{110})$ where $C_{6}$ is a six-dimensional D-vine copula, which is built via successive mixing from 15 bivariate linking copulas on levels. For parsimony, we use a one-truncated D-vine copula^27^ which has five parametric bivariate copulas $C(\cdot ;\theta_{101,011})$ , $C(\cdot ;\theta_{011,111})$ , $C(\cdot ;\theta_{111,100})$ , $C(\cdot ;\theta_{100,010})$ and $C(\cdot ;\theta_{010,110})$ that link $X_{101}$ with $X_{011}$ , $X_{011}$ with $X_{111}$ , $X_{111}$ with $X_{100}$ , $X_{100}$ with $X_{010}$ and $X_{010}$ with $X_{110}$ , respectively, in the first level of the vine and independence copulas in all the remaining levels of the vine (truncated after the first level). Figure 1 depicts the graphical representation of the one-truncated D-vine copula model. This truncation, as per the terminology by Brechmann et al.,^27^ offers a substantial reduction of the copula parameters. In our case there are 10 fewer bivariate copulas, which is extremely useful for estimation purposes given the typically small number of primary studies involved in meta-analysis.

Let $\theta =(\theta_{101,011},$ $\theta_{011,111},\theta_{111,100},\theta_{100,010},\theta_{010,110})$ be the copula parameter vector of the six-dimensional one-truncated D-vine copula and $c(u,v;\theta )=\frac{\partial C(u,v;\theta )}{\partial u\partial v}$ be a bivariate copula density. Then, the six-dimensional one-truncated D-vine copula density is decomposed in a simple manner by multiplying the bivariate copulas densities in the nodes of the tree in Figure 1, as indicated below

(1) $$\begin{array}{rl} & c_{6}(u_{101},u_{011},u_{111},u_{100},u_{010},u_{110};\theta )=c(u_{101},u_{011};\theta_{101,011})c(u_{011},u_{111};\theta_{011,111})\\ & \;\times c(u_{111},u_{100};\theta_{111,100})c(u_{100},u_{010};\theta_{100,010})c(u_{010},u_{110};\theta_{010,110}) \end{array}$$

Note that for a six-dimensional D-vine copula density there are $\frac{6!}{2}$ distinct permutations of the variables.^18^ To be concrete in the exposition of the theory, we use the permutation in Figure 1; the theory though also applies to the other permutations.

### 2.2. The multinomial one-truncated D-vine CMM with normal margins

The within-study model is the same as in the multinomial GLMM.^14^ That is

(2) $$\begin{array}{rl} & (Y_{i10d},Y_{i01d},Y_{i11d},Y_{i00d})|(X_{10d}=x_{10d},X_{01d}=x_{01d},X_{11d}=x_{11d})\\ & \;∼M_{4}(y_{i++d},l^{-1}(x_{10d},x_{01d},x_{11d}),l^{-1}(x_{01d},x_{10d},x_{11d}),l^{-1}(x_{11d},x_{10d},x_{01d})) \end{array}$$

where $M_{r}(n,$ $p_{1},\ldots ,p_{r-1})$ is shorthand notation for the multinomial distribution; r is the number of outcomes, n is the total number of diseased or non-diseased participants per single study, and $\left(p_{1},\ldots ,p_{r}\right)$ with $p_{r}=1-p_{1}-\ldots -p_{r-1}$ is the r -dimensional vector of success probabilities and $l^{-1}$ is the inverse multinomial logit link, for example, $l^{-1}(x_{10d},x_{01d},x_{11d})=\frac{e^{x_{10d}}}{1+e^{x_{10d}}+e^{x_{01d}}+e^{x_{11d}}}$ .

The stochastic representation of the between-studies model takes the form

(3) $$\begin{array}{rl} & (\Phi (X_{101};l(\pi_{101},\pi_{011},\pi_{111}),\sigma_{101}^{2}),\Phi (X_{011};l(\pi_{011},\pi_{101},\pi_{111}),\sigma_{011}^{2}),\Phi (X_{111};l(\pi_{111},\pi_{101},\pi_{011}),\sigma_{111}^{2})\\ & \Phi (X_{100};l(\pi_{100},\pi_{010},\pi_{110}),\sigma_{100}^{2}),\Phi (X_{010},l(\pi_{010},\pi_{100},\pi_{110}),\sigma_{010}^{2}),\Phi (X_{110};l(\pi_{110},\pi_{100},\pi_{010}),\sigma_{110}^{2}))∼C_{6}(\cdot ;\theta ) \end{array}$$

where $C_{6}(\cdot ;\theta )$ is a six-dimensional one-truncated D-vine copula with dependence parameter vector θ , Φ is the cumulative distribution function (cdf) of the N( $\mu ,\sigma^{2}$ ) distribution, and l is the multinomial logit link, for example, $l(\pi_{10d},\pi_{01d},\pi_{11d})=\log\;(\frac{\pi_{10d}}{1-\pi_{10d}-\pi_{01d}-\pi_{11d}})$ . The copula parameter vector θ contains parameters of the random effects model and they are separated from the univariate parameter vectors $\pi_{d}=(\pi_{10d},\pi_{01d},\pi_{11d})$ and $\sigma_{d}^{2}=(\sigma_{10d}^{2},\sigma_{01d}^{2},\sigma_{11d}^{2})$ . The $\pi_{d}$ ’s have the actual parameters of interest (see the notation of these parameters for each combination of test results in diseased and non-diseased participants in Table 2), since $\pi_{111}$ and $\pi_{110}$ is the meta-analytic parameter of joint TPF and joint FPF, respectively, and the meta-analytic parameters of the TPF ( d=1 ) or FPF ( d=0 ) for each test are functions of these parameters, viz.

(4) $$\pi_{1\cdot d}=\pi_{10d}+\pi_{11d}\;\pi_{\cdot 1d}=\pi_{01d}+\pi_{11d}$$

The univariate parameter vectors $\sigma_{d}^{2}$ ’s denote the variabilities of the random effects.

**Table 2.** Meta-analytic parameters of interest for each combination of test results in diseased and non-diseased participants in a 4×2 table.

		Disease (by gold standard)	
Test 1	Test 2	+	−
+	−	$\pi_{101}$	$\pi_{100}$
−	+	$\pi_{011}$	$\pi_{010}$
+	+	$\pi_{111}$	$\pi_{110}$
−	−	$1-\pi_{101}-\pi_{011}-\pi_{111}$	$1-\pi_{100}-\pi_{010}-\pi_{110}$

The models in (2) and (3) together specify a multinomial one-truncated D-vine CMM with joint likelihood

$$\begin{array}{rl} & L(\pi_{1},\pi_{0},\sigma_{1}^{2},\sigma_{0}^{2},\theta |y_{1},y_{0})=\prod_{i=1}^{N}\int_{[0,1{]}^{6}}\prod_{d=0}^{1}g(y_{i10d},y_{i01d},y_{i11d};y_{i++d},l^{-1}(x_{10d},x_{01d},x_{11d}),l^{-1}(x_{01d}\\ & x_{10d},x_{11d}),l^{-1}(x_{11d},x_{10d},x_{01d}))c_{6}(u_{101},u_{011},u_{111},u_{100},u_{010},u_{110};\theta )du_{101}du_{011}du_{111}du_{100}du_{010}du_{110} \end{array}$$

where

(5) $$\begin{array}{rl} & x_{10d}=\Phi^{-1}(u_{10d};l(\pi_{10d},\pi_{01t},\pi_{11d}),\sigma_{10d}^{2}),\;x_{01d}=\Phi^{-1}(u_{01d};l(\pi_{01d},\pi_{10d},\pi_{11d}),\sigma_{01d}^{2})\\ & x_{11d}=\Phi^{-1}(u_{11d};l(\pi_{11d},\pi_{10d},\pi_{01d}),\sigma_{11d}^{2}) \end{array}$$

#### 2.2.1. Relationship with the multinomial GLMM

In this section, we show what happens when all the bivariate copulas are bivariate normal (BVN) and the univariate distribution of the random effects is the $N(\mu ,\sigma^{2})$ distribution.

When all the bivariate pair-copulas are BVN copulas with correlation (copula) parameters $\rho_{101,011}$ , $\rho_{011,111}$ , $\rho_{111,100}$ , $\rho_{100,010}$ , $\rho_{010,110}$ , the resulting distribution is the six-variate normal with mean vector

$$\mu =(l(\pi_{101},\pi_{011},\pi_{111}),l(\pi_{011},\pi_{101},\pi_{111}),l(\pi_{111},\pi_{101},\pi_{111}),l(\pi_{100},\pi_{010},\pi_{110}),l(\pi_{010},\pi_{100},\pi_{110}),l(\pi_{110},\pi_{100},\pi_{110}))$$

and variance covariance matrix

$$\Sigma =\left(\begin{array}{cccccc} \sigma_{101}^{2}\\ \rho_{101,011}\sigma_{101}\sigma_{011} & \sigma_{011}^{2} & \\ \rho_{101,111}\sigma_{101}\sigma_{111} & \rho_{011,111}\sigma_{011}\sigma_{111} & \sigma_{111}^{2}\\ \rho_{101,100}\sigma_{101}\sigma_{100} & \rho_{011,100}\sigma_{011}\sigma_{100} & \rho_{111,100}\sigma_{111}\sigma_{100} & \sigma_{100}^{2}\\ \rho_{101,010}\sigma_{101}\sigma_{010} & \rho_{011,010}\sigma_{011}\sigma_{010} & \rho_{111,010}\sigma_{111}\sigma_{010} & \rho_{100,010}\sigma_{100}\sigma_{010} & \sigma_{010}^{2}\\ \rho_{101,110}\sigma_{101}\sigma_{110} & \rho_{011,110}\sigma_{011}\sigma_{110} & \rho_{111,110}\sigma_{111}\sigma_{110} & \rho_{100,110}\sigma_{100}\sigma_{110} & \rho_{010,110}\sigma_{010}\sigma_{110} & \sigma_{110}^{2} \end{array}\right)$$

where $\rho_{101,111}=\rho_{101,011}\rho_{011,111}$ , $\rho_{101,100}=\rho_{101,111}\rho_{111,100}$ , $\rho_{101,010}=\rho_{101,100}\rho_{010,110}$ , $\rho_{101,110}=\rho_{101,010}\rho_{010,110}$ , $\rho_{011,100}=\rho_{011,111}\rho_{111,100}$ , $\rho_{011,010}=\rho_{011,100}\rho_{100,010}$ , $\rho_{011,110}=\rho_{011,010}\rho_{010,110}$ , $\rho_{111,010}=\rho_{111,100}\rho_{100,010}$ , $\rho_{111,110}=\rho_{111,010}\rho_{010,110}$ and $\rho_{100,110}=\rho_{100,010}$ $\rho_{010,110}$ .

The covariance and correlation matrices as above play a central role in multivariate Gaussian structures. Nevertheless, two major difficulties in modelling such matrices are multidimensionality, as the number of parameters grows quadratically with dimension, and positive definiteness. Our approach overcomes both difficulties. Multidimensionality is controlled by focusing on a structured correlation matrix. As we use truncation, a structured correlation matrix is exploited and thus five instead of 15 dependence parameters have to be estimated, which is extremely useful as the sample size in our motivating example is so small ( N=11 ). In order to reduce the parameters even further, Trikalinos et al.^14^ proposed another structured variant by setting variances and correlations to be equal. Furthermore, our parametrisation of the six-variate Gaussian distribution as a one-truncated vine consists of algebraically independent correlations and avoids the positive definite constraints.^26^

Hence, our model includes the multinomial GLMM with a structured correlation matrix. Trikalinos et al.^14^ acknowledged that a more direct approach is to model the probabilities on the original scale in the form of a Dirichlet or multivariate beta distribution and leave this for future research. In the following section, we explicitly develop this method by using a one-truncated D-vine copula with beta margins representation of the multivariate beta distribution.

### 2.3. The multinomial one-truncated D-vine CMM with beta margins

Both the multinomial truncated D-vine CMM with normal margins and the multinomial GLMM assume the vector of probabilities for each combination of test results in diseased and non-diseased patients is on a transformed scale. However, by using a copula with beta margins representation of the random effects distribution, we can model the latent proportions on their original scale. As these proportions have unit sum constraints, we choose to elicit the random effects distribution over the conditional latent proportions that have algebraic independence using the transformation proposed by Wilson.^28^

The diseased and non-diseased subjects fall into four possible categories as indicated in the first two columns of Table 1. Assume that $X_{1}$ and $X_{0}$ represent the probability latent vectors for diseased and non-diseased subjects, respectively, falling into each category, given they have not fallen into any previous categories (rows). We can then recover the original latent proportions via

$$X_{10d}\;X_{01d}(1-X_{10d})\;X_{11d}(1-X_{01d})(1-X_{10d})\;(1-X_{11d})(1-X_{01d})(1-X_{10d})$$

Clearly, the latent proportions $X_{10d}$ remain on the original scale, but by permuting {10d,01d,11d} we can eventually get all the latent proportions on the original scale.

The within-study model takes the form

(6) $$\begin{array}{r} \left(Y_{i10d},Y_{i01d},Y_{11d},Y_{i00d})|(X_{10d}=x_{10d},X_{01d}=x_{01d},X_{11d}=x_{11d})∼M_{4}(y_{i++d},x_{10d},x_{01d}(1-x_{10d}),x_{11d}(1-x_{01d})(1-x_{10d})\right) \end{array}$$

The stochastic representation of the between-studies model is

(7) $$\begin{array}{rl} & (F\left(X_{101};\pi_{101},\gamma_{101}\right),F\left(X_{011};\frac{\pi_{011}}{1-\pi_{101}},\gamma_{011}\right),F\left(X_{111};\frac{\pi_{111}}{\left(1-\frac{\pi_{011}}{1-\pi_{101}}\right)(1-\pi_{101})},\gamma_{111}\right)\\ & F\left(X_{100};\pi_{100},\gamma_{100}\right),F\left(X_{010};\frac{\pi_{010}}{1-\pi_{100}},\gamma_{010}\right),F\left(X_{110};\frac{\pi_{110}}{\left(1-\frac{\pi_{010}}{1-\pi_{100}}\right)(1-\pi_{100})},\gamma_{110}\right))∼C_{6}(\cdot ;\theta ) \end{array}$$

where $C_{6}(\cdot ;\theta )$ is a six-dimensional one-truncated D-vine copula with dependence parameter vector θ and F(⋅;π,γ) is the cdf of the Beta( π,γ ) distribution with π the mean and γ the dispersion parameter. The copula parameter vector θ contains the dependence parameters of the random effects model and they are separated from the univariate parameters $\pi_{d}=(\pi_{10d},\pi_{01d},\pi_{11d})$ and $\gamma_{d}=(\gamma_{10d},\gamma_{01d},\gamma_{11d})$ . As in the preceding subsection, the $\pi_{d}$ ’s are the actual parameters of interest as the meta-analytic parameters of the TPF and FPF are functions of these parameters as shown in (4). The univariate parameter vectors $\gamma_{d}$ ’s denote the variabilities of the random effects.

The models in (6) and (7) together specify a multinomial one-truncated D-vine CMM with joint likelihood

$$\begin{array}{rl} & L(\pi_{1},\pi_{0},\gamma_{1},\gamma_{0},\theta |y_{1},y_{0})=\prod_{i=1}^{N}\int_{[0,1{]}^{6}}\prod_{d=0}^{1}g(y_{i10d},y_{i01d},y_{i11d};y_{i++d},x_{10d},x_{01d}(1-x_{10d}),\\ & x_{11d}(1-x_{01d})(1-x_{10d}))c_{6}(u_{101},u_{011},u_{111},u_{100},u_{010},u_{110};\theta )du_{101}du_{011}du_{111}du_{100}du_{010}du_{110}, \end{array}$$

where

(8) $$\begin{array}{rl} & x_{10d}=F^{-1}(u_{10d};\pi_{10d},\gamma_{10d}),\;x_{01d}=F^{-1}(u_{01d};\frac{\pi_{01d}}{1-\pi_{10d}},\gamma_{01d})\\ & x_{11d}=F^{-1}\left(u_{11d};\frac{\pi_{11d}}{\left(1-\frac{\pi_{01d}}{1-\pi_{10d}}\right)(1-\pi_{10d})},\gamma_{11d}\right) \end{array}$$

### 2.4. ML estimation and computational details

Estimation of the model parameters can be approached by the standard ML method, by maximising the logarithm of the joint likelihood. The estimated parameters can be obtained by using a quasi-Newton^29^ method applied to the logarithm of the joint likelihood. Motivated by our desire to use something like Newton’s method for its speed but without having to compute the Hessian matrix each time, we use a quasi-Newton method. Hence, the quasi-Newton minimisation with an input function the negative log-likelihood to be minimised, has output point of minimum and inverse Hessian at point of minimum.

For the multinomial one-truncated D-vine CMM, numerical evaluation of the joint pmf can be achieved with the following steps:

1. Calculate Gauss-Legendre^30^ quadrature points $\left\{u_{q}:q=1,\ldots ,N_{q}\right\}$ and weights $\left\{w_{q}:q=1,\ldots ,N_{q}\right\}$ in terms of standard uniform.
2. Convert from independent uniform random variables $\left\{u_{q_{101}}:q_{101}=1,\ldots ,N_{q}\right\}$ , $\left\{u_{q_{011}}:q_{011}=1,\ldots ,N_{q}\right\}$ , $\left\{u_{q_{111}}:q_{111}=1,\ldots ,N_{q}\right\}$ , $\left\{u_{q_{100}}:q_{100}=1,\ldots ,N_{q}\right\}$ , $\left\{u_{q_{010}}:q_{010}=1,\ldots ,N_{q}\right\}$ , and $\left\{u_{q_{110}}:q_{110}=1,\ldots ,N_{q}\right\}$ to dependent uniform random variables $v_{q_{101}},v_{q_{011}},v_{q_{111}}$ , $v_{q_{100}},v_{q_{010}}$ , and $v_{q_{110}}$ that have a one-truncated D-vine distribution $C_{6}(\cdot ;\theta )$ :
  **Table:**
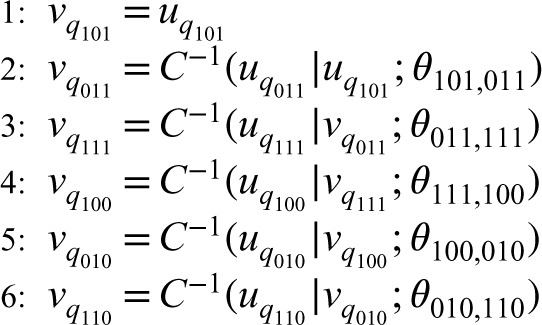
  where C(v|u;θ) and $C^{-1}(v|u;\theta )$ are conditional copula and inverse conditional copula cdfs, respectively. The method is based on the simulation algorithm of a one-truncated D-vine copula,^26^ where as input, instead of independent uniform variates, it uses the independent quadrature points.
3. Numerically evaluate the joint pmf, for example,
$$\begin{array}{rl} & \int_{[0,1{]}^{6}}\prod_{d=0}^{1}g(y_{i10d},y_{i01d},y_{i11d};y_{i++d},x_{10d},x_{01d}(1-x_{10d}),x_{11d}(1-x_{01d})(1-x_{10d}))\\ & \;\times c_{6}(u_{101},u_{011},u_{111},u_{100},u_{010},u_{110};\theta )du_{101}du_{011}du_{111}du_{100}du_{010}du_{110}, \end{array}$$
  with $x_{10d}$ , $x_{01d}$ and $x_{11d}$ as in (8), in a sextuple sum:
$$\begin{array}{rl} & \sum_{q_{101}=1}^{N_{q}}\sum_{q_{011}=1}^{N_{q}}\sum_{q_{111}=1}^{N_{q}}\sum_{q_{100}=1}^{N_{q}}\sum_{q_{010}=1}^{N_{q}}\sum_{q_{110}=1}^{N_{q}}w_{q_{101}}w_{q_{011}}w_{q_{111}}w_{q_{100}}w_{q_{010}}w_{q_{110}}\prod_{d=0}^{1}g(y_{i10d},y_{i01d},y_{i11d};\\ & y_{i++d},x_{10d},x_{q_{01d}}\left(1-x_{q_{10d}}\right),x_{q_{11d}}\left(1-x_{q_{01d}}\right)\left(1-x_{q_{10d}}\right)) \end{array}$$
  where
$$\begin{array}{rl} & x_{q_{10d}}=F^{-1}(v_{q_{10d}};\pi_{10d},\gamma_{10d}),\;x_{q_{01d}}=F^{-1}\left(v_{q_{01d}};\frac{\pi_{01d}}{1-\pi_{10d}},\gamma_{01d}\right)\\ & x_{q_{11d}}=F^{-1}\left(v_{q_{11d}};\frac{\pi_{11d}}{\left(1-\frac{\pi_{01d}}{1-\pi_{10d}}\right)(1-\pi_{10d})},\gamma_{11d}\right) \end{array}$$

With Gauss-Legendre quadrature, the same nodes and weights are used for different functions; this helps in yielding smooth numerical derivatives for numerical optimisation via quasi-Newton.

## 3. Small-sample efficiency – Misspecification of the random effects distribution

In this section, we study the small-sample efficiency and robustness of the ML estimation of the multinomial one-truncated D-vine CMM. In Section 3.1, we investigate the dependence structure misspecification by using the multinomial D-vine CMM without truncation as the true model. In Section 3.2, we gauge the small-sample efficiency of the ML method and in Section 2.4, we investigate the misspecification of either the parametric margin or bivariate copula of the random effects distribution.

We use the following simulation process:

1. Simulate $\left(u_{101},u_{011},u_{111},u_{100},u_{010},u_{110}\right)$ from a six-variate (one-truncated) D-vine distribution $C_{6}(\cdot ;\theta )$ .
2. Convert to normal or beta realisations $x_{10d}$ , $x_{01d}$ and $x_{11d}$ via the relations in (5) or (8), respectively.
3. Simulate the size of diseased and non-diseased subjects $n_{1}$ and $n_{0}$ , respectively, from a shifted gamma distribution to obtain heterogeneous study sizes,^31^ that is,
$$n_{d}∼\mathrm{sGamma}(\alpha =1.2,\beta =0.01,\mathrm{lag}=30)$$
  and round off $n_{1}$ and $n_{0}$ to the nearest integer.
4.
  - For normal margins draw $\left(y_{10d},y_{01d},y_{11d},y_{00d}\right)$ from
$$M_{4}(n_{d},l^{-1}(x_{10d},x_{01d},x_{11d}),l^{-1}(x_{01d},x_{10d},x_{11d}),l^{-1}(x_{11d},x_{10d},x_{01d}))$$
  - For beta margins draw $\left(y_{10d},y_{01d},y_{11d},y_{00d}\right)$ from
$$M_{4}(n_{d},x_{10d},x_{01d}(1-x_{10d}),x_{11d}(1-x_{01d})(1-x_{10d}))$$

In our simulations, we set the sample size and the true univariate and dependence parameters to mimic the data on N=11 studies from the systematic review that examines the screening accuracy of shortened humerus and shortened femur of the fetus markers^14^ and investigate five simulation scenarios. These are complemented with five additional simulation scenarios that can be found online in the Supplemental Material. Therein, the true univariate and dependence parameters are set to either mimic the data on N=22 studies obtained from a meta-analysis that aims to determine whether anticyclic citrullinated peptide antibody identifies more accurately patients with rheumatoid arthritis than does rheumatoid factor,^32^ or to larger values of TPF/FPF and dependence than the ones in the aforementioned meta-analyses. When we mimic these data, we prefer to use N=11 studies but as true parameters the ones obtained from fitting the multinomial one-truncated D-vine CMM to the data from the N=22 studies.

In line with our previous contributions in CMMs,^8,12,25,33–37^ we use bivariate parametric copulas with different tail dependence behaviour, namely the BVN with intermediate tail dependence, Frank with tail independence, and Clayton with positive lower tail dependence. For the latter, we also use its rotated versions to provide negative upper-lower tail dependence (Clayton rotated by 90 ∘ ), positive upper tail dependence (Clayton rotated by 180 ∘ ) and negative lower-upper tail dependence (Clayton rotated by 270 ∘ ). To make it easier to compare strengths of dependence, we convert the BVN, Frank and rotated Clayton estimated copula parameters to Kendall’s τ ’s in (−1,1) via the following relations^38–40^:

(9) $$\begin{array}{rl} \tau & =\frac{2}{\pi }\arcsin(\theta ) \end{array}$$

(10) $$\begin{array}{rl} \tau & =\left\{ \begin{array}{cc} 1-4\theta^{-1}-4\theta^{-2}\int_{\theta }^{0}\frac{t}{e^{t}-1}dt, & \theta <0\\ 1-4\theta^{-1}+4\theta^{-2}\int_{0}^{\theta }\frac{t}{e^{t}-1}dt, & \theta >0 \end{array}\right. \end{array}$$

(11) $$\begin{array}{rl} \tau & =\left\{ \begin{array}{l} \theta /(\theta +2),\;\mathrm{by}\;0\circ \;\mathrm{or}\;180\circ \\ -\theta /(\theta +2),\;\mathrm{by}\;90\circ \;\mathrm{or}\;270\circ \end{array}\right. \end{array}$$

In Supplemental Figure 1, to depict the different directions of tail dependence, we show contour plots of the Clayton and its rotated copulas with standard normal margins and dependence parameters corresponding to a Kendall’s τ value of 0.5 on absolute value. Sharper corners (relative to ellipse) indicate tail dependence in one of the four tails. We refer the interested reader to Section 4 by Nikoloulopoulos^8^ for more details on tail dependence and how a copula with tail dependence differs from a BVN copula.

### 3.1. Structure misspecification – Sensitivity analysis to one-truncation

We have simulated from the multinomial (non-truncated) D-vine CMM with BVN copulas and normal margins, that is, the multinomial GLMM with an unstructured correlation matrix. The true univariate parameters are $\pi_{1}=\{0.044,0.091,0.299\}$ , $\pi_{0}=\{0.017,$ 0.049,0.030} , $\sigma_{1}=\{1.493,0.607,$ 0.563} , $\sigma_{0}=\{0.917,0.455,0.576\}$ , the five correlation parameters (converted to Kendall’s τ ) are {−0.518,0.560,0.266,−0.039,0.506} and the 10 additional conditional correlation parameters (converted to Kendall’s τ ) are {0.128,−0.432,0.372,0.804,0.299, 0.927,−0.205,0.348,−0.346,0.950} . We obtain the ML estimates of the multinomial one-truncated D-vine CMM with BVN copulas and normal margins, that is, the multinomial GLMM with a structured correlation matrix and also include in the comparison the estimates of the bivariate GLMM^6^ from two separate meta-analyses, one for each test, for the common meta-analytic parameters $\pi_{1\cdot d}$ and $\pi_{\cdot 1d}$ of TPF ( d=1 ) or FPF ( d=0 ) for each test.

Table 3 (Supplemental Table 1) contains the resultant mean biases, root mean square errors (RMSEs) and standard deviations (SDs), along with the square roots of the average theoretical variances ( $\sqrt{\bar{V}}$ ), scaled by 100, for the ML estimates of the multinomial GLMM with a structured correlation matrix and the separate bivariate GLMMs. The theoretical variance of the ML estimates for each simulated dataset is obtained via the gradients and the Hessian computed numerically during the quasi-Newton minimisation.

**Table 3.** Small sample of sizes N=11 simulations ( $10^{3}$ replications, $N_{q}=15$ ) from the multinomial D-vine CMM with BVN copulas and normal margins (that is the multinomial GLMM with an unstructured correlation matrix) and mean biases, RMSEs and SDs, along with the square roots of the average theoretical variances ( $\sqrt{\bar{V}}$ ), scaled by 100, for the ML estimates of the multinomial one-truncated D-vine CMM with BVN copulas and normal margins (i.e. the multinomial GLMM with a structured correlation matrix) and the bivariate GLMMs from two separate meta-analyses, one for each test, for the common meta-analytic parameters $\pi_{1\cdot d}$ and $\pi_{\cdot 1d}$ of TPF ( d=1 ) or FPF ( d=0 ) for each test.

	Multinomial one-truncated D-vine CMM				Separate bivariate GLMMs			
	with BVN copulas and normal margins							
True values	Bias	SD	$\sqrt{\bar{V}}$	RMSE	Bias	SD	$\sqrt{\bar{V}}$	RMSE
$\pi_{101}=0.044$	−0.168	2.818	1.635	2.823	–	–	–	–
$\pi_{011}=0.091$	0.381	2.175	1.839	2.208	–	–	–	–
$\pi_{111}=0.299$	0.392	4.531	3.335	4.548	–	–	–	–
$\pi_{100}=0.017$	0.104	0.727	0.656	0.734	–	–	–	–
$\pi_{010}=0.049$	0.028	0.957	0.897	0.958	–	–	–	–
$\pi_{110}=0.030$	−0.014	0.737	0.645	0.737	–	–	–	–
$\pi_{1\cdot 1}=0.342$	0.225	3.908	3.040	3.915	4.031	3.880	3.354	5.595
$\pi_{\cdot 11}=0.390$	0.774	5.616	4.035	5.669	−1.597	5.141	4.077	5.383
$\pi_{1\cdot 0}=0.047$	0.090	1.179	0.944	1.183	0.260	1.152	1.075	1.180
$\pi_{\cdot 10}=0.079$	0.014	1.395	1.176	1.396	0.102	1.326	1.225	1.330
$\sigma_{101}=1.493$	−12.300	47.008	28.229	48.591	–	–	–	–
$\sigma_{011}=0.607$	8.286	32.276	23.396	33.323	–	–	–	–
$\sigma_{111}=0.563$	−4.155	21.626	13.883	22.022	–	–	–	–
$\sigma_{100}=0.917$	−11.584	36.732	38.157	38.516	–	–	–	–
$\sigma_{010}=0.455$	−4.339	23.531	23.064	23.928	–	–	–	–
$\sigma_{110}=0.576$	−7.728	27.271	23.955	28.345	–	–	–	–
$\tau_{101,011}=-0.518$	3.779	31.133	31.243	31.361	–	–	–	–
$\tau_{011,111}=0.560$	−6.437	30.600	27.609	31.270	–	–	–	–
$\tau_{111,100}=0.266$	1.420	39.642	37.824	39.667	–	–		–
$\tau_{100,010}=-0.039$	14.107	47.553	50.795	49.601	–	–	–	–
$\tau_{010,110}=0.506$	1.561	40.204	65.686	40.235	–	–	–	–

CMM: copula mixed model; RMSEs: root mean square errors; SDs: standard deviations; GLMM: generalised linear mixed model; BVN: bivariate normal; TPF: true positive fraction; FPF: false positive fraction; ML: maximum likelihood.

$N_{q}$ is the number of quadrature points and weights.

The simulation results from Table 3 and Supplemental Table 1 show that the multinomial one-truncated D-vine CMM leads to unbiased and efficient estimates when the assumption of conditional independence (truncation) is violated. The use of an unstructured correlation matrix is not a distributional concern about the dependence between the tests and makes no difference other than introducing more dependence parameters than are actually required. This is due to the main result by Joe et al.^41^: all the bivariate margins of the vine copula have (tail) dependence if the bivariate copulas at level 1 have (tail) dependence. It is also revealed that assuming independence between the two tests by fitting two separate meta-analyses, might lead to biased estimates ${\hat{\pi }}_{1\cdot d}$ and ${\hat{\pi }}_{\cdot 1d}$ of the meta-analytic parameters of TPF ( d=1 ) or FPF ( d=0 ) for each test as the between tests information is neglected.

### 3.2. Margin and bivariate copula misspecification

In this subsection, a simulation study with four different scenarios is conducted to (a) assess the performance of the ML method, and (b) investigate the effect of the misspecification of either the parametric margin or bivariate copula of the random effects distribution.

In the first scenario, the simulated data are generated from a multinomial one-truncated D-vine CMM with BVN copulas and normal margins (the resulting model is the same with the multinomial GLMM), while in the second scenario the simulated data are generated from a multinomial one-truncated D-vine CMM with BVN copulas and beta margins. Table 4 (Supplemental Table 2) contains the resultant mean biases, RMSEs and SDs, along with $\sqrt{\bar{V}}$ , scaled by 100, for the ML estimates of the multinomial truncated D-vine CMM with BVN copulas and normal margins, that is, the multinomial GLMM. We simulate from normal margins and estimated with normals margins (left side of Table 4 and Supplemental Table 2) or simulate from beta margins and estimated with normals margins (right side of Table 4 and Supplemental Table 2).

**Table 4.** Small sample of sizes N=11 simulations ( $10^{3}$ replications, $N_{q}=15$ ) from the multinomial one-truncated D-vine copula mixed model with BVN copulas and both normal (that is the same with the multinomial GLMM) and beta margins and mean biases, RMSEs and SDs, along with the square roots of the average theoretical variances ( $\sqrt{\bar{V}}$ ), scaled by 100, for the MLEs of the multinomial one-truncated D-vine copula mixed model with BVN copulas and normal margins (multinomial GLMM).

True (simulated) bivariate copula: BVN									
True (simulated) univariate margin: normal^a^					True (simulated) univariate margin: beta				
True values	Bias	SD	$\sqrt{\bar{V}}$	RMSE	True values	Bias	SD	$\sqrt{\bar{V}}$	RMSE
$\pi_{101}=0.037$	0.103	4.184	1.611	4.185	$\pi_{101}=0.091$	−6.181	2.787	1.529	6.781
$\pi_{011}=0.093$	0.449	2.125	1.929	2.172	$\pi_{011}=0.086$	0.773	2.154	2.164	2.289
$\pi_{111}=0.295$	0.671	4.769	3.685	4.816	$\pi_{111}=0.292$	1.716	4.445	3.615	4.765
$\pi_{100}=0.017$	0.128	0.753	0.673	0.764	$\pi_{100}=0.024$	−0.461	0.724	0.616	0.858
$\pi_{010}=0.049$	0.067	0.995	0.915	0.997	$\pi_{010}=0.054$	−0.357	1.014	0.874	1.075
$\pi_{110}=0.030$	0.026	0.744	0.653	0.745	$\pi_{110}=0.034$	−0.336	0.724	0.637	0.798
$\pi_{1\cdot 1}=0.331$	0.774	4.232	3.364	4.303	$\pi_{1\cdot 1}=0.383$	−4.465	4.204	3.444	6.133
$\pi_{\cdot 11}=0.388$	1.121	5.898	4.422	6.003	$\pi_{\cdot 11}=0.378$	2.489	5.476	4.444	6.015
$\pi_{1\cdot 0}=0.047$	0.154	1.064	0.927	1.075	$\pi_{1\cdot 0}=0.058$	−0.797	1.014	0.881	1.290
$\pi_{\cdot 10}=0.079$	0.093	1.443	1.204	1.446	$\pi_{\cdot 10}=0.088$	−0.693	1.430	1.157	1.589
$\sigma_{101}=1.699$	−16.076	59.052	30.366	61.201	$\gamma_{101}=0.186$	–	60.137	38.151	–
$\sigma_{011}=0.543$	13.386	36.956	21.917	39.306	$\gamma_{011}=0.016$	–	36.311	27.041	–
$\sigma_{111}=0.585$	−3.872	23.458	15.216	23.775	$\gamma_{111}=0.066$	–	20.195	15.013	–
$\sigma_{100}=0.929$	−9.645	37.941	37.035	39.148	$\gamma_{100}=0.015$	–	35.120	35.450	–
$\sigma_{010}=0.490$	−6.043	23.022	21.888	23.802	$\gamma_{010}=0.011$	–	22.394	20.545	–
$\sigma_{110}=0.570$	−8.459	27.213	22.832	28.497	$\gamma_{110}=0.010$	–	26.296	23.279	
$\tau_{101,011}=-0.525$	5.066	31.093	32.432	31.503	$\tau_{101,011}=-0.525$	16.168	31.319	29.155	35.246
$\tau_{011,111}=0.558$	−5.571	31.463	27.902	31.952	$\tau_{011,111}=0.300$	15.472	29.684	29.010	33.474
$\tau_{111,100}=0.185$	0.800	37.174	36.929	37.183	$\tau_{111,100}=0.197$	2.185	39.347	38.178	39.408
$\tau_{100,010}=0.022$	4.256	41.838	55.494	42.053	$\tau_{100,010}=-0.029$	7.018	44.426	56.755	44.977
$\tau_{010,110}=0.576$	−7.875	42.820	72.731	43.538	$\tau_{010,110}=0.544$	−2.784	40.708	64.822	40.803

BVN: bivariate normal; GLMM: generalised linear mixed model; RMSEs: root mean square errors; SDs: standard deviations; MLEs: maximum likelihood estimations; TPF: true positive fraction; FPF: false positive fraction.

^a^The resulting model is the same as the multinomial GLMM; $\pi_{1\cdot d}$ and $\pi_{\cdot 1d}$ are the meta-analytic parameters of TPF ( d=1 ) or FPF ( d=0 ) for each test; $\pi_{11d}$ is the meta-analytic parameter of the joint TPF ( d=1 ) or joint FPF ( d=0 ); $N_{q}$ is the number of quadrature points and weights.

In the third scenario, the simulated data are generated from a multinomial one-truncated D-vine CMM with normal margins and

$$\mathrm{Cln}\{0\circ ,90\circ \}=\left\{ \begin{array}{lcc} \text{Clayton rotated by}\;0\circ & \text{if} & \tau >0\\ \text{Clayton rotated by}\;90\circ & \text{if} & \tau <0 \end{array}\right.$$

copulas, while in the fourth scenario the simulated data are generated from a multinomial one-truncated D-vine CMM with beta margins and Cln{0∘,90∘} copulas. Table 5 (Supplemental Table 3) contains the resultant mean biases, RMSEs, and SDs, along with $\sqrt{\bar{V}}$ , scaled by 100, for the ML estimates of the multinomial one-truncated D-vine CMM with BVN copulas and beta margins. We simulate from normal margins and estimated with beta margins (left side of Table 5 and Supplemental Table 3) or simulate from beta margins and estimated with beta margins (right side of Table 5 and Supplemental Table 3). That is, the simulation results in the left side of the table are from the misspecification of both the margin and bivariate copula, while the simulation results in the right side of the table are from the misspecification of the parametric bivariate copula only.

**Table 5.** Small sample of sizes N=11 simulations ( $10^{3}$ replications, $N_{q}=15$ ) from the multinomial one-truncated D-vine copula mixed model with Cln{0∘,90∘} copulas and both normal and beta margins and mean biases, RMSEs and SDs, along with the square roots of the average theoretical variances ( $\sqrt{\bar{V}}$ ), scaled by 100, for the MLEs of the multinomial one-truncated D-vine copula mixed model with BVN copulas and beta margins.

True (simulated) bivariate copula: Cln{0∘,90∘}									
True (simulated) univariate margin: normal					True (simulated) univariate margin: beta				
	Bias	SD	$\sqrt{\bar{V}}$	RMSE		Bias	SD	$\sqrt{\bar{V}}$	RMSE
$\pi_{101}=0.037$	4.493	4.004	1.606	6.018	$\pi_{101}=0.091$	−0.562	2.903	1.981	2.957
$\pi_{011}=0.093$	0.217	1.700	1.277	1.714	$\pi_{011}=0.086$	0.222	1.380	1.253	1.397
$\pi_{111}=0.295$	−0.330	3.574	3.320	3.589	$\pi_{111}=0.292$	0.162	3.292	3.287	3.296
$\pi_{100}=0.017$	0.805	1.120	0.824	1.380	$\pi_{100}=0.024$	−0.030	0.696	0.697	0.696
$\pi_{010}=0.049$	0.425	1.036	0.922	1.120	$\pi_{010}=0.054$	0.023	0.946	0.941	0.946
$\pi_{110}=0.030$	0.414	0.881	0.719	0.973	$\pi_{110}=0.034$	−0.005	0.704	0.714	0.704
$\pi_{1\cdot 1}=0.331$	4.163	3.286	3.089	5.304	$\pi_{1\cdot 1}=0.383$	−0.400	3.221	3.283	3.246
$\pi_{\cdot 11}=0.388$	−0.113	4.426	3.924	4.428	$\pi_{\cdot 11}=0.378$	0.384	3.913	3.747	3.932
$\pi_{1\cdot 0}=0.047$	1.219	1.527	1.100	1.954	$\pi_{1\cdot 0}=0.058$	−0.035	0.954	0.988	0.954
$\pi_{\cdot 10}=0.079$	0.839	1.566	1.311	1.776	$\pi_{\cdot 10}=0.088$	0.018	1.333	1.316	1.333
$\sigma_{101}=1.699$	–	7.300	4.179	–	$\gamma_{101}=0.186$	−1.778	6.815	5.378	7.043
$\sigma_{011}=0.543$	–	1.528	1.053	–	$\gamma_{011}=0.016$	−0.173	1.073	1.045	1.087
$\sigma_{111}=0.585$	–	2.677	2.946	–	$\gamma_{111}=0.066$	−0.656	2.681	3.164	2.760
$\sigma_{100}=0.929$	–	1.817	1.451	–	$\gamma_{100}=0.015$	−0.101	1.152	1.206	1.157
$\sigma_{010}=0.490$	–	1.025	0.905	–	$\gamma_{010}=0.011$	−0.078	0.882	1.072	0.886
$\sigma_{110}=0.570$	–	1.141	0.861	–	$\gamma_{110}=0.010$	−0.055	0.877	0.894	0.879
$\tau_{101,011}=-0.525$	−12.089	24.950	34.488	27.724	$\tau_{101,011}=-0.525$	−9.396	25.705	36.629	27.368
$\tau_{011,111}=0.558$	−5.753	27.463	25.334	28.059	$\tau_{011,111}=0.300$	3.714	30.593	26.617	30.817
$\tau_{111,100}=0.185$	2.300	30.430	33.248	30.517	$\tau_{111,100}=0.197$	4.809	32.575	36.717	32.928
$\tau_{100,010}=0.022$	−1.069	34.234	47.512	34.251	$\tau_{100,010}=-0.029$	−2.164	38.512	47.589	38.573
$\tau_{010,110}=0.576$	5.097	24.725	106.770	25.245	$\tau_{010,110}=0.544$	10.771	24.970	92.963	27.194

RMSEs: root mean square errors; SDs: standard deviations; MLEs: maximum likelihood estimations; BVN: bivariate normal; TPF: true positive fraction; FPF: false positive fraction.

$\pi_{1\cdot d}$ and $\pi_{\cdot 1d}$ are the meta-analytic parameters of TPF ( d=1 ) or FPF ( d=0 ) for each test; $\pi_{11d}$ is the meta-analytic parameter of the joint TPF ( d=1 ) or joint FPF ( d=0 ); $N_{q}$ is the number of quadrature points and weights.

Conclusions from the values in Tables 4 and 5 and Supplemental Tables 2 and 3 are the following:

- ML with the true multinomial one-truncated D-vine CMM is highly efficient according to the calculated biases, SDs and RMSEs. For example, in Table 4 (Supplemental Table 2) where the true univariate margins are normal the scaled biases for the ML estimates (MLEs) of $\pi_{0}$ for the multinomial one-truncated vine CMM with BVN copulas and normal margins range from 0.026 ( −0.028 ) to 0.128 ( 0.034 ).
- The MLEs of the univariate parameters of main interest $\pi_{1},\pi_{0}$ and their functions, that is, the meta-analytic parameters $\pi_{1\cdot d}$ and $\pi_{\cdot 1d}$ of TPF ( d=1 ) or FPF ( d=0 ) for each test, are not robust to margin misspecification, for example, in Table 4 (Supplemental Table 2) where the true univariate margins are beta, the scaled biases for the MLEs of $\pi_{1}$ for the multinomial one-truncated vine CMM with BVN copulas and normal margins range from −6.181 ( −1.074 ) to 1.716 ( 1.362 ).
- The MLEs of τ ’s, that is, the dependencies of the random effects, are robust to margin misspecification, for example, in Table 4 (Supplemental Table 2) where the true univariate margins are beta the scaled biases for the MLEs of τ ’s for the multinomial one-truncated vine CMM with BVN copulas and normal margins range from −2.784 ( −9.982 ) to 16.168 ( 9.042 ).
- The MLEs of the univariate parameters of main interest $\pi_{1},\pi_{0}$ and their functions $\pi_{1\cdot t},\pi_{\cdot 1t}$ , that is, the meta-analytic parameters $\pi_{1\cdot t}$ and $\pi_{\cdot 1t}$ of TPF ( d=1 ) or FPF ( d=0 ) for each test, are reasonably robust to bivariate copula misspecification. For example, in Table 5 (Supplemental Table 3) where the true bivariate copulas are Cln{0∘,90∘} the scaled biases for the MLEs of $\pi_{0}$ for the multinomial one-truncated vine CMM with BVN copulas and beta margins range from −0.030 ( −0.098 ) to 0.023 ( 0.287 ).
- The MLEs of $\gamma_{1},\gamma_{0}$ or $\sigma_{1},\sigma_{0}$ , that is, the variabilities of the random effects, are reasonably robust to bivariate copula misspecification. For example, in Table 5 (Supplemental Table 3) where the true bivariate copulas are Cln{0∘,90∘} the scaled biases for the MLEs of $\gamma_{0}$ for the multinomial one-truncated vine CMM with BVN copulas and beta margins range from −0.101 ( −1.123 ) to −0.055 ( −0.644 ).
- The MLEs of τ ’s, that is, the dependencies of the random effects, are robust to bivariate copula misspecification, for example, in Table 5 (Supplemental Table 3) where the true bivariate copulas are Cln{0∘,90∘} the scaled biases of $\hat{\tau }$ ’s for the multinomial one-truncated vine CMM with BVN copulas and beta margins range from −9.396 ( −4.439 ) to 10.771 ( 1.968 ).

From the summaries above, we observe that a small sample size (i.e. the number of studies) introduces larger biases, SDs and $\sqrt{\bar{V}}$ ’s for the Kendall’s τ and variability parameters $\gamma_{1},\gamma_{0}$ (beta margins) or $\sigma_{1},\sigma_{0}$ (normal margins). This is because six variability and five Kendall’s τ parameters have to be estimated in addition to the six probability parameters that are of main interest. Trikalinos et al.^14^ also acknowledged these parameters are often not well estimated for small sample sizes. Nevertheless, this does not have implications for the parameters of main interest $\pi_{1},\pi_{0}$ and their functions, that is, the meta-analytic parameters $\pi_{1\cdot d}$ and $\pi_{\cdot 1d}$ of TPF ( d=1 ) or FPF ( d=0 ) for each test.

Furthermore, the simulation results indicate that the effect of misspecifying the marginal choice can be seen as substantial for both the univariate parameters of main interest $\pi_{1},\pi_{0}$ and their functions, that is, the meta-analytic parameters $\pi_{1\cdot d}$ and $\pi_{\cdot 1d}$ of TPF ( d=1 ) or FPF ( d=0 ) for each test. Hence, the multinomial GLMM can lead to biased meta-analytic estimates of interest $\pi_{1},\pi_{0}$ and their functions, that is the meta-analytic parameters $\pi_{1\cdot d}$ and $\pi_{\cdot 1d}$ of TPF ( d=1 ) or FPF ( d=0 ) for each test, as it is restricted to a normal margin specification. We also show that the effect of misspecifying the copula choice can be seen as minimal for both the univariate parameters and Kendall’s tau, which is a strictly increasing function of the copula parameter for any pair-copula, as (a) the meta-analytic parameters are a univariate inference, and hence, it is the univariate marginal distribution that matters and not the type of the pair-copula, and (b) Kendall’s tau only accounts for the dependence dominated by the middle of the data, and it is expected to be similar amongst different families of bivariate copulas. However, the tail dependence varies, and is a property to consider when choosing amongst different families of bivariate copulas. Any inference that depends on the joint distribution will essentially show the effects of different model (random effect distribution) assumptions such as the pair-copula choice. We discuss such an inference in the forthcoming section.

## 4. Summary receiver operating characteristic curves

Though typically the focus of meta-analysis has been to derive the summary-effect estimates, there is increasing interest in alternative summary outputs, such as summary receiver operating characteristic (SROC) curves. Trikalinos et al.^14^ have not derived the SROC curves from the multinomial GLMM, as the latent vector of probabilities of each combination of test results in diseased and non-diseased patients is on a transformed scale via the multinomial logit link.

In this section, we derive the SROC curves from the multinomial one-truncated D-vine CMM with beta margins, taking advantage of the fact that some of the latent proportions can be on the original scale. We have to first strategically permute the variables as $X_{11d},X_{10d},X_{01d}$ , so that $X_{111}$ (latent joint TPF) and $X_{110}$ (latent joint FPF) are on the original scale. Hence, the within-study and between-studies models take the form

$$\begin{array}{rl} & (Y_{11d},Y_{i10d},Y_{i01d},Y_{i00d})|(X_{11d}=x_{11d},X_{10d}=x_{10d},X_{01d}=x_{01d})\\ & \;∼M_{4}(y_{i++d},x_{11d},x_{10d}(1-x_{11d}),x_{01d}(1-x_{10d})(1-x_{11d})) \end{array}$$

and

$$\begin{array}{rl} & (F(X_{111};\pi_{111},\gamma_{111}),F(X_{110};\pi_{110},\gamma_{110}),F(X_{101};\frac{\pi_{101}}{1-\pi_{111}},\gamma_{101}),F(X_{011};\frac{\pi_{011}}{\left(1-\frac{\pi_{101}}{1-\pi_{111}}\right)(1-\pi_{111})},\gamma_{011}),\\ & F(X_{100};\frac{\pi_{100}}{1-\pi_{110}},\gamma_{100}),F(X_{010};\frac{\pi_{010}}{\left(1-\frac{\pi_{100}}{1-\pi_{110}}\right)(1-\pi_{110})},\gamma_{010}))∼C_{6}(\cdot ;\theta^{\prime }) \end{array}$$

respectively, where $\theta^{\prime }=(\theta_{111,110},\theta_{110,101},\theta_{101,011},\theta_{011,100},\theta_{100,010})$ . With this permutation, we achieve that $X_{11d}∼\text{Beta}(\pi_{11d},\gamma_{11d})$ and the bivariate copula that links $X_{111}$ and $X_{110}$ is $C(F(X_{111};\pi_{111},\gamma_{111}),$ $F(X_{110};\pi_{110},\gamma_{110});\theta_{111,110})$ .

We use the notion of median regression of $X_{111}$ (latent joint TPF) on $X_{110}$ (latent joint FPF) to derive the SROC curve.^8^ For $x_{110}$ in range of $X_{110}$ , let $x_{111}:={\tilde{x}}_{111}(x_{110})$ denote a solution to the equation $\mathrm{Pr}(X_{111}\leq x_{111}|X_{110}=x_{110})=0.5.$ Then the scatter plot of ${\tilde{x}}_{111}(x_{110})$ and $x_{110}$ is the median regression curve of $X_{111}$ on $X_{110}$ . In addition to just using just median ( q=0.5 ) regression curves, we will also exploit the use of quantile regression curves with a focus on high ( q=0.99 ) and low quantiles ( q=0.01 ) which are strongly associated with the upper and lower tail dependence imposed from each parametric family of copulas. These can be seen as confidence regions, as per the terminology by Rucker and Schumacher,^42^ of the median regression curve. For q∈{0.01,0.5,0.99} to find the quantile regression curves:

1. Set $C(u_{111}|u_{110};\theta_{111,110})=q$ ;
2. Solve for the quantile regression curve $u_{111}:={\tilde{u}}_{111}(u_{110},q;\theta_{111,110})=C^{-1}(q|u_{110};\theta_{111,110})$ ;
3. Replace $u_{11d}$ by $F(x_{11d};\pi_{11d},\gamma_{11d})$ ;
4. Plot $x_{111}:={\tilde{x}}_{111}(x_{110},q)$ versus $x_{110}$ .

As there is no a-priori reason to regress $X_{111}$ (latent joint TPF) on $X_{110}$ (latent joint FPF) instead of the other way around,^43^ quantile regression curves of $X_{110}$ on $X_{111}$ are also derived in a similar manner. Finally, in order to reserve the nature of a bivariate response instead of a univariate response along with a covariate, we plot the corresponding contour graph of the bivariate copula density with beta margins. The contour plot can be seen as the predictive region (analogously to Reitsma et al.^5^) of the estimated pair $\left({\hat{\pi }}_{111},{\hat{\pi }}_{110}\right)$ of the meta-analytic parameters of joint TPF and joint FPF.

## 5. Joint meta-analysis of shortened humerus and shortened femur of the fetus markers

In the research area of detecting fetuses with Down’s syndrome, many screening accuracy of second-trimester ultrasound markers have been developed. Down syndrome is the most common clinical significant chromosomal abnormality among fetuses.^44^ There has been a substantial interest in the prenatal detection of affected fetuses so that parents can be prepared for the birth of an affected child or even consider pregnancy termination.^45^ Mothers and fetuses identified by a positive screening test result are typically offered a definitive diagnosis via amniocentesis, an invasive diagnostic test.^14^

We demonstrate the modelling process of the proposed approach by insightfully re-analysing the data on N=11 studies from the systematic review that examines the screening accuracy of shortened humerus and shortened femur of the fetus markers (two out of seven ultrasonographic markers or their combination in detecting Down syndrome by Smith-Bindman et al.^45^). These data have previously been analysed by Trikalinos et al.^14^ who fitted the multinomial GLMM and are shown in Supplemental Table 4. Note in passing that the multinomial GLMM is a special case of our model when all the bivariate copulas are BVN and the univariate distribution of the random effects is the $N(\mu ,\sigma^{2})$ distribution as shown in Section 2.2.1.

### 5.1. Modelling process

We fit the multinomial one-truncated D-vine CMM for all different pair copulas and univariate marginal distributions. We use the decomposition of the vine copula density in (1), as different decompositions will lead to similar results due to the small sample size.^25^ In our general statistical model, there are no constraints in the choice of the parametric marginal or pair-copula distributions. This is one of the limitations of the multinomial GLMM where all the pair copulas are BVN and marginal distributions are normal. However, for ease of interpretation, we do not mix pair-copulas or margins. To make it easier to compare strengths of dependence amongst different copulas, we convert from the BVN, Frank and (rotated) Clayton θ ’s to τ ’s via the relations in (9) to (11), respectively. In cases when fitting the multinomial one-truncated D-vine CMM, the resultant estimate of one of the Kendall’s τ parameters was close to the right ( 0.95 ) or left boundary ( −0.95 ) of its parameter space, we set the corresponding bivariate copula to comonotonic (Fréchet lower bound) or countermonotonic (Fréchet lower bound) copula, respectively. For the Clayton and Clayton rotated by 180 ∘ (Clayton rotated by 90 ∘ and Clayton rotated by 270 ∘ ) as they interpolate from the independence when θ→0 to the comonotonic copula when θ→∞ (interpolate from the countermonotonic copula when θ→−∞ to the independence when θ→0 ) we substitute the BVN copula that interpolates from the Fréchet lower (perfect negative dependence) to the Fréchet upper (perfect positive dependence) bound when the Kendall’s τ parameters are close to independence.

To find the model that provides the best fit, we don’t use goodness-of-fit procedures; but rather we use the log-likelihood at the maximum likelihood estimate as a rough diagnostic measure for goodness of fit between the models. The goodness-of-fit procedures involve a global distance measure between the model-based and empirical distribution, hence they might not be sensitive to tail behaviours and are not diagnostic in the sense of suggesting improved parametric models in the case of small p -values.^26^ For vine copulas, Dissmann et al.^46^ find that pair-copula selection based on likelihood seem to be better than using bivariate goodness-of-fit tests. A larger likelihood value indicates a model that better approximates both the dependence structure of the data and the strength of dependence in the tails.

### 5.2. Results

The maximised log-likelihoods, estimates and standard errors from fitting the multinomial one-truncated D-vine CMM with normal and beta margins are given in Supplemental Tables 5 and 6, respectively. The log-likelihoods show that a multinomial one-truncated D-vine CMM with beta margins and Cln{0∘,90∘} bivariate copulas provides the best fit. Note that as there exists counter-monotonic dependence among $X_{101}$ and $X_{011}$ ( $\tau_{101,011}=-0.95$ ), this model coincides with the model with

$$\text{Cln}\{0\circ ,270\circ \}=\left\{ \begin{array}{lcc} \text{Clayton rotated by}\;0\circ & \text{if} & \tau >0\\ \text{Clayton rotated by}\;270\circ & \text{if} & \tau <0 \end{array}\right.$$

bivariate copulas and beta margins as both the Clayton copula rotated by 90∘ and the Clayton copula rotated by 270∘ go to their limiting case the counter-monotonic copula. It is revealed that a multinomial one-truncated D-vine CMM with the vector of probabilities of each combination of tests results in diseased and non-diseased patients on the original scale provides better fit than the multinomial GLMM, which models the vector of probabilities of each combination of tests results in diseased and non-diseased patients on a transformed scale. The improvement over the multinomial GLMM is small in terms of the likelihood principle, but for a sample size such as N=11 , −3190.43−(−3192.90)=2.5 units log-likelihood difference is sufficient.

The fact that the best-fitting bivariate copulas are Clayton reveals that there exists lower tail dependence amongst the latent vector of probabilities of each combination of tests results in diseased and non-diseased patients. It is also apparent that the estimates of the meta-analytic parameters of interest from the multinomial one-truncated vine CMMs with normal margins (Supplemental Table 5) differentiate from the ones with beta margins (Supplemental Table 6). For example, the resultant meta-analytic estimate ${\hat{\pi }}_{1\cdot 1}$ of TPF for shortened humerous ranges from 0.331 to 0.343 and from 0.382 to 0.391 in Supplemental Tables 5 and 6, respectively. This is consistent with the simulation results and conclusions in Section 3. The main parameters of interest, that is, the meta-analytic parameters $\pi_{1\cdot d}$ and $\pi_{\cdot 1d}$ of TPF ( d=1 ) or FPF ( d=0 ) for each test and the meta-analytic parameters of the joint TPF $\pi_{111}$ and joint FPF $\pi_{110}$ , are biased when the univariate random effects are misspecified. Our general model can allow both normal and beta margins, that is, it is not restricted to normal margins as the multinomial GLMM.

In order to reveal if the use of the proposed model is worthy, when standard bivariate analyses from two separate meta-analyses are easy, we also fit the bivariate copula mixed model with both beta and normal margins and different bivariate copulas to each of the tests ignoring the within-study information. According to the likelihood principle, a bivariate copula mixed model with a Clayton copula and beta margins provides the best fit for both markers (Supplemental Table 7). It is apparent that the meta-analytic estimates of TPF ( ${\hat{\pi }}_{1\cdot 1}=0.391,{\hat{\pi }}_{\cdot 11}=0.385$ ) from the selected multinomial one-truncated vine CMM, that is, the one with Cln {0∘,90∘} copulas and beta margins, differentiate from the ones ( ${\hat{\pi }}_{1\cdot 1}=0.375,{\hat{\pi }}_{\cdot 11}=0.375$ ) from the selected separate bivariate CMMs, that is, the ones with Clayton copula and beta margins. The meta-analytic estimates of TPF from the separate bivariate analyses are underestimated because the between tests information is neglected. This is consistent with the simulation results and conclusions in Section 3.

To compare the TPFs and FPFs of shortened humerus and shortened femur, we use the difference between the estimated meta-analytic parameters of TPF ( ${\hat{\pi }}_{\cdot 11}-{\hat{\pi }}_{1\cdot 1}$ ) or FPF ( ${\hat{\pi }}_{\cdot 10}-{\hat{\pi }}_{1\cdot 0}$ ) of the tests (shortened femur minus shortened humerus). A positive difference in, for example, the TPF favours shortened femur, in that its average TPF is higher than that of shortened humerous. A difference of zero favours neither test, and a negative difference favours shortened humerous. For FPF, the direction is reversed, for example, a negative difference favours shortened femur. Table 6 shows the differences (shortened femur minus shortened humerus) along with the corresponding standard errors, 95% confidence intervals (CI), z -statistics and p -values. The differences in FPFs are generally similar across analyses and favour shortened humerous. The TPFs do not differ across analyses beyond what is expected by chance. Nevertheless, the point estimates of the differences reveal that in the multinomial GLMM analysis the TPF favours shortened femur, in the multinomial one-truncated D-vine CMM with Cln {0∘,90∘} copulas and beta margins analysis the TPF favours shortened humerous, and in the separate bivariate CMMs analyses the TPF favours neither test. Note in passing the confidence intervals are wider for the GLMM analysis as the covariances of the meta-analytic parameters are zero due to the assumed independence among the tests.

**Table 6.** Comparative test performance: Differences (shortened femur minus shortened humerus) in the summary TPFs or FPFs along with the corresponding standard errors, 95% confidence intervals (CI), z -statistics and p -values.

Difference in TPR			95/% CI			
$\pi_{\cdot 11}-\pi_{1\cdot 1}$	Est.	SE	Lower	Upper	z	p -value
Multinomial one-truncated D-vine CMM	0.056	0.042	−0.027	0.139	1.329	0.184
with BVN copulas and normal margins^a^						
Multinomial one-truncated D-vine CMM	−0.005	0.058	−0.119	0.108	−0.093	0.926
with Cln {0∘,90∘} copulas and beta margins						
Separate bivariate CMMs	0.000	0.058	−0.113	0.113	0.001	0.999
with Clayton copulas and beta margins						
Difference in FPR			95/% CI			
$\pi_{\cdot 10}-\pi_{1\cdot 0}$	Est.	SE	Lower	Upper	z	p -value
Multinomial one-truncated D-vine CMM	0.032	0.009	0.014	0.050	3.423	0.001
with BVN copulas and normal margins^a^						
Multinomial one-truncated D-vine CMM	0.027	0.009	0.010	0.045	3.100	0.002
with Cln {0∘,90∘} copulas and beta margins						
Separate bivariate CMMs	0.026	0.013	0.000	0.052	1.971	0.049
with Clayton copulas and beta margins						

TPF: true positive fraction; FPF: false positive fraction; CMM: copula mixed model; BVN: bivariate normal; GLMM: generalised linear mixed model; SE: square error.

^a^The resulting model is the same as the multinomial GLMM.

### 5.3. SROC curves

**Figure 2.**
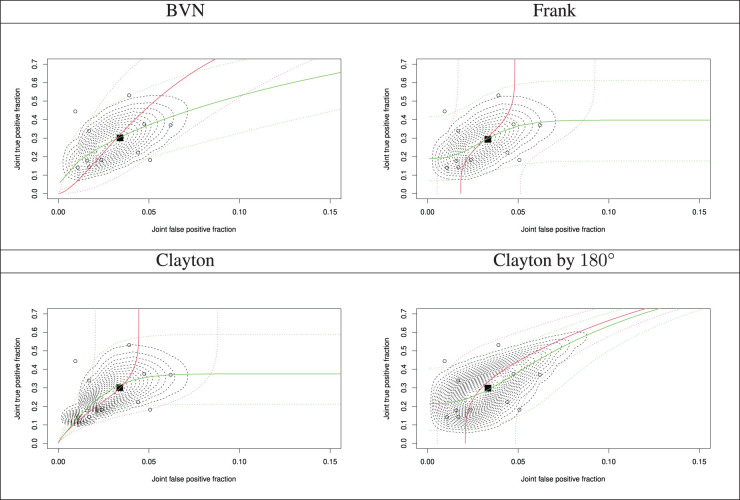
Summary receiver operating characteristic (SROC) curves with a predictive region and summary operating points (a pair of the model-based joint true positive fraction and joint false positive fraction) from the fitted multinomial one-truncated D-vine copula mixed models with beta margins along with the study estimates. ◼ : summary point $\left({\hat{\pi }}_{111},{\hat{\pi }}_{110}\right)$ of the estimated pair of the meta-analytic parameters of the joint true positive fraction and joint false positive fraction; ∘ : individual study estimate; red and green lines represent the quantile regression curves $x_{111}:={\tilde{x}}_{111}(x_{110},q)$ and $x_{110}:={\tilde{x}}_{110}(x_{111},q)$ , respectively; for q=0.5 solid lines and for q∈{0.01,0.99} dotted lines (confidence region).

Furthermore, assuming independence between the tests, the performance of the markers in combination is not performed and estimates of the joint accuracy measures are not derived. It also affects the joint tail probabilities of the joint accuracy measures, and hence, prediction through the SROC curves, since the dependence parameter between the latent joint TPF $X_{111}$ and joint FPF $X_{110}$ affects the shape of the SROC curve and this is set to independence. Figure 2 demonstrates the SROC curves with a confidence region and summary operating points (a pair of the estimated meta-analytic parameters of the joint TPF ${\hat{\pi }}_{111}$ and joint FPF ${\hat{\pi }}_{110}$ ; shown by the black square) from all the multinomial one-truncated D-vine CMMs with beta margins, along with the study estimates (shown by the circles in Figure 2). Sharper corners in the predictive region indicate tail dependence. For all the graphs the joint TPF and joint FPF at study i (point estimates) have been calculated as

$$\frac{y_{i111}}{y_{i++1}}\;\text{and}\;\frac{y_{i110}}{y_{i++0}}$$

respectively, and the estimated parameters by refitting the models using the permutation in Section 4. The estimated Kendall’s τ association between the latent joint TPF $X_{111}$ and joint FPF $X_{110}$ is roughly ${\hat{\tau }}_{111,110}=0.45$ from all fitted copulas, but the shapes and regions of the SROCs are distinct as parametric bivariate copulas have varying tail behaviour. The predictive region from the best fitted copula (Clayton) has a sharper corner at the lower tail, as the Clayton copula has lower tail dependence.

## 6. Discussion

We have proposed a multinomial one-truncated D-vine CMM for joint meta-analysis and comparison of two diagnostic tests on the same participants in a paired design with a gold standard. Our model generalises the multinomial GLMM^14^ that can lead to biased estimates of the meta-analytic parameters of interest. It essentially provides an improvement over the multinomial GLMM as the random effects distribution is expressed via a vine copula that allows for flexible dependence modelling, different from assuming simple linear correlation structures and normality. This strength of multivariate meta-analysis approaches that use copulas has been pointed out.^47,48^ Vine copulas, by choosing bivariate copulas appropriately, can have a flexible range of lower/upper tail dependence.^41^ The multinomial one-truncated D-vine CMM allows for selection of parametric bivariate copulas and univariate margins independently among a variety of parametric families. Hence, the latent probabilities of each combination of test results in diseased and non-diseased patients can be modelled on the original proportions scale and can be tail dependent.

Ignoring the fact that the same individuals receive both tests, that is, fitting a separate meta-analysis for each test can lead to biased estimates of the meta-analytic parameters of TPF and FPF for each test as the within-study dependence is neglected. Furthermore, assuming independence between the tests, it will affect the joint tail probabilities, and hence, prediction through the SROC curves, since the dependence parameter between the latent joint TPF and joint FPF affects the shape of the SROC curve and this is set to independence. In an era of evidence-based medicine, decision makers need procedures, such as the SROC curves, to make predictions. For the multinomial one-truncated D-vine CMM with beta margins, we derived the associated SROC curves. The SROC curves essentially show the effects of different model assumptions, such as the choice of parametric bivariate copula and its tail dependence properties, because they are inferences that depend on the joint distribution. Our proposed model with normal margins or the multinomial GLMM^14^ cannot be used to produce the SROC curves, since the latent proportions are modelled on a transformed scale via the multinomial logit link.

We propose an efficient ML estimation technique based on dependent Gauss-Legendre quadrature points that have a one-truncated D-vine copula distribution. We use the notion of a truncated at level 1 vine copula that leads to a substantial reduction of the dependence parameters. This is extremely useful for estimation purposes given the typical small sample sizes in meta-analysis of diagnostic test accuracy studies. Trikalinos et al.^14^ estimated the multinomial GLMM using Markov chain Monte Carlo methods in the Bayesian framework and acknowledge that optimising the likelihood for joint meta-analysis is non-trivial, because it involves calculating complicated integrals numerically. Our numerical method that is based on dependent Gauss-Legendre quadrature points, that have an one-truncated D-vine copula distribution, successively computes the six-dimensional integrals in sextuple sums over the dependent quadrature points and weights.

Authors of primary studies of diagnostic accuracy that assess two tests with paired designs where each test is applied to the same patients should not only report details of each index test under investigation, but also describe how the index tests were compared to each other, that is, report the data as separate 4×2 tables as in Table 1. However, existing guidance^49^ for reporting diagnostic studies has no specific instructions for comparative accuracy studies. Vali et al.^50^ assessed the reporting of information on test comparisons in comparative accuracy studies and examine whether data for the construction of 4×2 tables were reported by paired accuracy studies. This was the case in only a handful of paired studies. This is also acknowledged in the Cochrane Handbook for Systematic Reviews of Diagnostic Test Accuracy; Deeks et al.^51^ stressed that many comparative accuracy studies with a paired test design do not present results in a 4×2 table but rather give a separate 2×2 table of the results of each index test against the gold standard. This illustrates a clear need for improvement in the standards of reporting for comparative accuracy studies given the fact that we have now proposed the machinery to meta-analyse comparative studies with a paired test design. Nevertheless, the proposed model or the multinomial GLMM^14^ that both consider the case the test results are cross-classified cannot be extended to compare the accuracy of more than two tests as the number of model parameters increases rapidly. For example, one needs $2(2^{T}-1)$ parameters, where T is the number of tests, to only model the probabilities of each combination of tests results in diseased and non-diseased patients. Nikoloulopoulos,^37^ without using the information on the agreement between the tests, proposes a one-factor CMM that can be used for conducting meta-analysis of comparative accuracy studies with three or more tests.

## Software

R functions to derive estimates and simulate from the multinomial one-truncated D-vine CMM for meta-analysis of two diagnostic tests accounting for within and between studies dependence are part of the R package **CopulaREMADA**.^52^ The data and code used in Section 5 are given as code examples in the package.

## Supplemental Material

sj-pdf-1-smm-10.1177_09622802241269645 - Supplemental material for Joint meta-analysis of two diagnostic tests accounting for within and between studies dependence Supplemental material, sj-pdf-1-smm-10.1177_09622802241269645 for Joint meta-analysis of two diagnostic tests accounting for within and between studies dependence by Aristidis K Nikoloulopoulos in Statistical Methods in Medical Research
