# Supplementary material for: Joint meta-analysis of two diagnostic tests accounting for within and between studies dependence
Source: Stat Methods Med Res. 2024 Sep 11;33(10):1800–17. doi: 10.1177/09622802241269645 (PMC11577699; doi:10.1177/09622802241269645)
Supplement: sj-pdf-1-smm-10.1177_09622802241269645 - Supplemental material for Joint meta-analysis of two diagnostic tests accounting for within and between studies dependence [file sj-pdf-1-smm-10.1177_09622802241269645.pdf]

Supplementary Material for

Joint meta-analysis of two diagnostic tests accounting for within and between studies dependence

by

Aristidis K. Nikoloulopoulos *

*a.nikoloulopoulos@uea.ac.uk, School of Mathematics, University of East Anglia, Norwich NR4 7TJ, U.K.

Supplementary Figure 1: Contour plots of Clayton and its rotated copulas with standard normal margins and dependence parameters corresponding to a Kendall's τ value of .5 in absolute value.

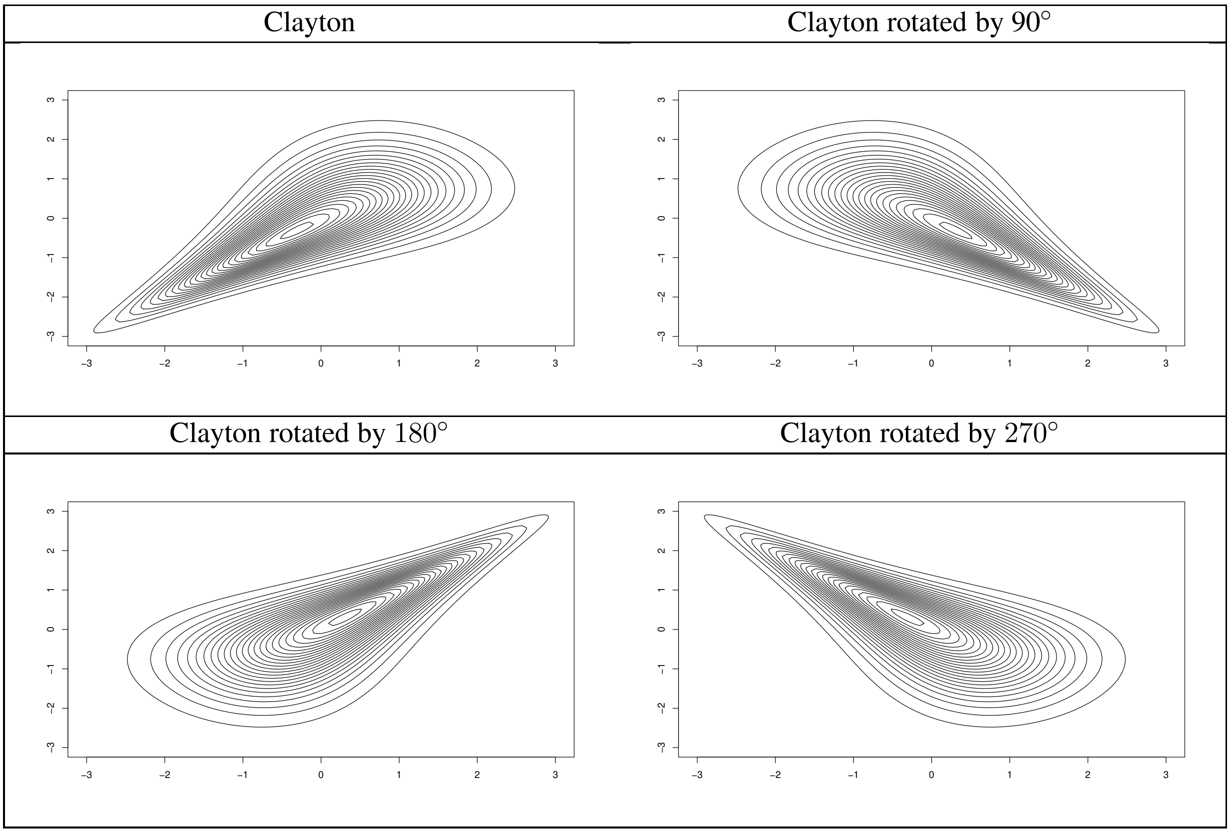

Supplementary Table 1: Small sample of sizes $N = 11$ simulations (10^3 replications, $N_q = 15$) from the multinomial D-vine copula mixed model with BVN copulas and normal margins (that is the multinomial GLMM with an unstructured correlation matrix) and mean biases, root mean square errors (RMSEs) and standard deviations (SDs), along with the square roots of the average theoretical variances ($\sqrt{\bar{V}}$), scaled by 100, for the ML estimates of the multinomial 1-truncated D-vine copula mixed model with BVN copulas and normal margins (that is the multinomial GLMM with a structured correlation matrix) and the bivariate GLMMs from two separate meta-analyses, one for each test, for the common meta-analytic parameters $\pi_{1\cdot d}$ and $\pi_{\cdot 1d}$ of TPF ($d = 1$) or FPF ($d = 0$) for each test.

	Multinomial truncated D-vine CMM				Separate bivariate GLMMs			
True values	Bias	SD	$\sqrt{\bar{V}}$	RMSE	Bias	SD	$\sqrt{\bar{V}}$	RMSE
$\pi_{101} = 0.127$	0.088	2.744	2.534	2.746	-	-	-	-
$\pi_{011} = 0.038$	0.142	1.092	1.043	1.102	-	-	-	-
$\pi_{111} = 0.804$	-0.28	2.63	2.403	2.645	-	-	-	-
$\pi_{100} = 0.151$	-0.153	3.503	3.299	3.506	-	-	-	-
$\pi_{010} = 0.121$	0.214	3.511	2.948	3.517	-	-	-	-
$\pi_{110} = 0.079$	-0.099	1.815	1.739	1.818	-	-	-	-
$\pi_{1\cdot 1} = 0.931$	-0.192	1.543	1.175	1.555	-0.273	1.131	1.085	1.164
$\pi_{\cdot 11} = 0.842$	-0.138	2.782	2.631	2.785	-0.452	2.707	2.363	2.745
$\pi_{1\cdot 0} = 0.230$	-0.252	4.464	3.652	4.472	-0.487	4.223	3.786	4.251
$\pi_{\cdot 10} = 0.200$	0.115	4.335	3.753	4.336	0.512	4.057	3.459	4.09
$\sigma_{101} = 0.549$	1.968	26.301	21.518	26.374	-	-	-	-
$\sigma_{011} = 0.680$	-1.23	29.814	31.805	29.84	-	-	-	-
$\sigma_{111} = 0.327$	-4.118	20.682	17.956	21.088	-	-	-	-
$\sigma_{100} = 0.900$	-3.774	24.99	22.792	25.274	-	-	-	-
$\sigma_{010} = 0.995$	-5.279	27.208	24.166	27.716	-	-	-	-
$\sigma_{110} = 0.860$	-8.614	26.141	22.341	27.523	-	-	-	-
$\tau_{101,011} = -0.368$	12.261	53.303	60.567	54.695	-	-	-	-
$\tau_{011,111} = 0.281$	17.77	46.858	53.856	50.114	-	-	-	-
$\tau_{111,100} = 0.198$	-17.442	47.525	37.091	50.625	-	-	-	-
$\tau_{100,010} = 0.316$	3.073	26.728	20.029	26.904	-	-	-	-
$\tau_{010,110} = 0.397$	3.853	27.561	22.364	27.829	-	-	-	-

The 10 true additional conditional correlation parameters (converted to Kendall's τ) are $\{0.164, -0.246, 0.2741, 0.462, 0.313, 0.304, 0.732, 0.076, 0.95, -0.209\}$; N_q is the number of quadrature points and weights.

Supplementary Table 2: Small sample of sizes $N = 11$ simulations (10^3 replications, $N_q = 15$) from the multinomial 1-truncated D-vine copula mixed model with BVN copulas and both normal (that is the same with the multinomial GLMM) and beta margins and mean biases, root mean square errors (RMSEs) and standard deviations (SDs), along with the square roots of the average theoretical variances ($\sqrt{V}$), scaled by 100, for the MLEs of the multinomial 1-truncated D-vine copula mixed model with BVN copulas and normal margins (multinomial GLMM).

True (simulated) bivariate copula: BVN									
True (simulated) univariate margin: normal*					True (simulated) univariate margin: beta				
$\pi_{101} = 0.136$	0.104	2.502	2.260	2.505	$\pi_{101} = 0.146$	-1.074	2.741	2.686	2.944
$\pi_{011} = 0.037$	0.057	0.839	0.783	0.841	$\pi_{011} = 0.041$	-0.362	0.934	0.989	1.002
$\pi_{111} = 0.795$	-0.182	2.160	1.952	2.167	$\pi_{111} = 0.781$	1.362	2.319	2.246	2.689
$\pi_{100} = 0.152$	-0.006	3.712	3.359	3.712	$\pi_{100} = 0.166$	-1.059	4.520	4.157	4.643
$\pi_{010} = 0.114$	-0.028	2.889	2.731	2.889	$\pi_{010} = 0.144$	-2.072	3.939	3.412	4.451
$\pi_{110} = 0.076$	0.034	1.955	1.843	1.955	$\pi_{110} = 0.091$	-1.325	2.067	1.962	2.455
$\pi_{1\cdot 1} = 0.931$	-0.078	1.085	1.017	1.088	$\pi_{1\cdot 1} = 0.928$	0.287	1.105	1.160	1.142
$\pi_{\cdot 11} = 0.833$	-0.125	2.415	2.196	2.418	$\pi_{\cdot 11} = 0.822$	0.999	2.695	2.646	2.875
$\pi_{1\cdot 0} = 0.229$	0.028	4.041	3.767	4.041	$\pi_{1\cdot 0} = 0.257$	-2.384	4.448	4.223	5.047
$\pi_{\cdot 10} = 0.190$	0.005	3.955	3.778	3.955	$\pi_{\cdot 10} = 0.236$	-3.398	5.048	4.435	6.084
$\sigma_{101} = 0.644$	-5.502	18.325	22.611	19.133	$\gamma_{101} = 0.037$	-	15.169	25.131	-
$\sigma_{011} = 0.345$	-3.638	18.887	25.737	19.234	$\gamma_{011} = 0.011$	-	15.876	34.346	-
$\sigma_{111} = 0.174$	-2.872	12.706	18.513	13.027	$\gamma_{111} = 0.001$	-	11.019	18.986	-
$\sigma_{100} = 0.896$	-5.278	19.610	23.388	20.308	$\gamma_{100} = 0.069$	-	15.812	29.605	-
$\sigma_{010} = 0.967$	-5.911	21.670	25.063	22.462	$\gamma_{010} = 0.101$	-	17.328	30.091	-
$\sigma_{110} = 0.884$	-6.729	21.199	24.987	22.241	$\gamma_{110} = 0.071$	-	16.480	27.681	-
$\tau_{101,011} = -0.578$	-10.075	23.547	48.396	25.612	$\tau_{101,011} = -0.472$	-9.982	19.470	48.194	21.880
$\tau_{011,111} = -0.677$	-1.105	21.450	64.405	21.479	$\tau_{011,111} = -0.558$	-1.730	19.565	47.952	19.641
$\tau_{111,100} = 0.454$	10.040	29.108	40.193	30.791	$\tau_{111,100} = 0.743$	1.604	14.261	56.995	14.350
$\tau_{100,010} = 0.322$	2.286	21.972	21.762	22.091	$\tau_{100,010} = 0.089$	9.042	16.138	32.059	18.499
$\tau_{010,110} = 0.445$	3.949	22.071	22.901	22.421	$\tau_{010,110} = 0.371$	5.454	16.933	26.766	17.789

*: The resulting model is the same as the multinomial GLMM; $\pi_{1\cdot d}$ and $\pi_{\cdot 1d}$ are the meta-analytic parameters of TPF ($d = 1$) or FPF ($d = 0$) for each test; π_{11d} is the meta-analytic parameter of the joint TPF ($d = 1$) or joint FPF ($d = 0$); N_q is the number of quadrature points and weights.

Supplementary Table 3: Small sample of sizes $N = 11$ simulations (10^3 replications, $N_q = 15$) from the multinomial 1-truncated D-vine copula mixed model with Cln$\{0°, 90°\}$ copulas and both normal and beta margins and mean biases, root mean square errors (RMSEs) and standard deviations (SDs), along with the square roots of the average theoretical variances ($\sqrt{V}$), scaled by 100, for the MLEs of the multinomial 1-truncated D-vine copula mixed model with BVN copulas and beta margins.

True (simulated) bivariate copula: Cln$\{0°, 90°\}$									
True (simulated) univariate margin: normal					True (simulated) univariate margin: beta				
	Bias	SD	$\sqrt{V}$	RMSE		Bias	SD	$\sqrt{V}$	RMSE
$\pi_{101} = 0.1$	5.020	4.464	2.254	6.718	$\pi_{101} = 0.1$	-0.425	3.016	1.978	3.046
$\pi_{011} = 0.2$	-0.348	3.496	1.833	3.513	$\pi_{011} = 0.2$	0.495	4.795	3.697	4.820
$\pi_{111} = 0.6$	-5.136	4.728	2.729	6.981	$\pi_{111} = 0.6$	0.050	2.801	2.254	2.801
$\pi_{100} = 0.1$	3.601	3.549	3.151	5.056	$\pi_{100} = 0.1$	0.287	3.675	2.952	3.687
$\pi_{010} = 0.2$	0.991	4.695	4.166	4.799	$\pi_{010} = 0.2$	-0.098	4.783	3.569	4.784
$\pi_{110} = 0.1$	2.695	3.232	2.771	4.209	$\pi_{110} = 0.1$	0.200	3.088	2.432	3.095
$\pi_{1\cdot 1} = 0.7$	-0.116	3.068	2.002	3.070	$\pi_{1\cdot 1} = 0.7$	-0.375	3.003	2.485	3.026
$\pi_{\cdot 11} = 0.8$	-5.484	5.852	3.340	8.019	$\pi_{\cdot 11} = 0.8$	0.546	5.653	3.713	5.679
$\pi_{1\cdot 0} = 0.2$	6.296	5.781	5.369	8.548	$\pi_{1\cdot 0} = 0.2$	0.486	6.210	5.013	6.229
$\pi_{\cdot 10} = 0.3$	3.687	3.272	2.668	4.929	$\pi_{\cdot 10} = 0.3$	0.102	3.236	2.297	3.237
$\sigma_{101} = 1$	-	8.127	3.492	-	$\gamma_{101} = 0.1$	-1.503	3.814	2.941	4.099
$\sigma_{011} = 1$	-	4.018	1.738	-	$\gamma_{011} = 0.1$	-0.948	4.232	3.425	4.337
$\sigma_{111} = 1$	-	4.327	3.753	-	$\gamma_{111} = 0.1$	-1.471	3.851	3.477	4.122
$\sigma_{100} = 1$	-	4.857	4.213	-	$\gamma_{100} = 0.1$	-0.832	4.697	4.339	4.770
$\sigma_{010} = 1$	-	5.635	5.206	-	$\gamma_{010} = 0.1$	-1.123	3.986	3.702	4.142
$\sigma_{110} = 1$	-	5.827	4.791	-	$\gamma_{110} = 0.1$	-0.644	4.994	4.386	5.036
$\tau_{101,011} = -0.8$	1.747	18.818	19.445	18.899	$\tau_{101,011} = -0.8$	-4.439	10.480	16.630	11.381
$\tau_{011,111} = 0.8$	-7.954	21.997	19.858	23.391	$\tau_{011,111} = 0.8$	1.968	11.673	16.082	11.838
$\tau_{111,100} = 0.8$	-3.242	11.900	16.804	12.334	$\tau_{111,100} = 0.8$	-0.946	11.270	16.818	11.310
$\tau_{100,010} = -0.8$	2.604	11.814	14.771	12.098	$\tau_{100,010} = -0.8$	-0.562	12.480	15.568	12.493
$\tau_{010,110} = -0.8$	0.066	10.805	16.605	10.805	$\tau_{010,110} = -0.8$	-0.280	11.650	17.026	11.653

$\pi_{1\cdot d}$ and $\pi_{\cdot 1d}$ are the meta-analytic parameters of TPF ($d = 1$) or FPF ($d = 0$) for each test; π_{11d} is the meta-analytic parameter of the joint TPF ($d = 1$) or joint FPF ($d = 0$); N_q is the number of quadrature points and weights.

Supplementary Table 4: Cross-classification of counts of shortened femur or shortened humerus among infants with trisomy 21 and healthy infants.

Study	y_{i001}	y_{i011}	y_{i101}	y_{i111}	y_{i000}	y_{i010}	y_{i100}	y_{i110}
Benacerraf et al. (1991)	11	1	3	9	354	21	6	19
Benacerraf et al. (1992)	9	6	0	17	514	40	11	23
Benacerraf et al. (1994)	23	2	0	20	100	3	2	1
Biagiotti et al. (1994)	14	3	0	10	411	29	29	31
Bromley et al. (1997)	25	7	3	18	161	11	2	3
Johnson et al. (1995)	21	7	0	8	662	92	5	35
Lockwood et al. (1993)	30	0	6	6	4602	78	111	83
Nyberg et al. (1993)	31	3	3	8	871	29	27	15
Nyberg et al. (1998)	105	10	7	20	886	33	1	10
Rodis et al. (1991)	4	0	5	2	1357	39	39	35
Vintzileos et al. (1996)	11	1	6	4	419	25	24	25

Supplementary Table 5: Maximized log-likelihoods, estimates and standard errors (SE) of the multinomial 1-truncated D-vine copula mixed models with normal margins for the accuracy of shortened humerus and shortened femur of the fetus in detecting Down syndrome in liveborn infants.

	BVN*		Frank		Cln$\{0°, 90°\}$		Cln$\{0°, 270°\}$		Cln$\{180°, 90°\}$		Cln$\{180°, 270°\}$	
	Est.	SE	Est.	SE	Est.	SE	Est.	SE	Est.	SE	Est.	SE
π_{101}	0.037	0.026	0.034	0.024	0.035	0.026	0.037	0.026	0.035	0.025	0.036	0.025
π_{011}	0.093	0.026	0.094	0.024	0.083	0.023	0.103	0.017	0.082	0.022	0.089	0.025
π_{111}	0.295	0.048	0.299	0.047	0.308	0.044	0.305	0.043	0.304	0.047	0.301	0.047
π_{100}	0.017	0.006	0.017	0.006	0.017	0.006	0.017	0.006	0.017	0.006	0.017	0.006
π_{010}	0.049	0.007	0.046	0.006	0.047	0.006	0.047	0.006	0.047	0.008	0.047	0.008
π_{110}	0.030	0.006	0.029	0.005	0.030	0.005	0.030	0.005	0.029	0.005	0.029	0.005
$\pi_{1\cdot 1}$	0.331	0.042	0.333	0.041	0.343	0.043	0.342	0.038	0.339	0.041	0.337	0.040
$\pi_{\cdot 11}$	0.388	0.057	0.393	0.055	0.390	0.049	0.408	0.049	0.386	0.054	0.390	0.054
$\pi_{1\cdot 0}$	0.047	0.008	0.046	0.007	0.047	0.008	0.048	0.008	0.046	0.007	0.046	0.007
$\pi_{\cdot 10}$	0.079	0.011	0.075	0.009	0.077	0.010	0.077	0.010	0.076	0.010	0.076	0.010
σ_{101}	1.699	0.670	1.670	0.688	1.757	0.663	1.683	0.673	1.700	0.661	1.681	0.667
σ_{011}	0.543	0.303	0.522	0.287	0.608	0.281	0.336	0.177	0.662	0.283	0.562	0.296
σ_{111}	0.585	0.189	0.578	0.186	0.496	0.172	0.498	0.156	0.557	0.177	0.550	0.169
σ_{100}	0.929	0.266	0.939	0.243	0.935	0.267	0.924	0.255	0.928	0.267	0.928	0.267
σ_{010}	0.490	0.104	0.411	0.075	0.400	0.067	0.398	0.066	0.513	0.112	0.513	0.112
σ_{110}	0.570	0.160	0.521	0.144	0.534	0.151	0.532	0.150	0.552	0.154	0.552	0.154
$\tau_{101,011}$	-0.525	0.480	-0.531	0.440	-0.699	0.553	-0.352	0.304	-0.771	0.488	-0.652	0.498
$\tau_{011,111}$	0.558	0.458	0.572	0.458	0.278	0.372 ‡	0.95	-	0.430	0.267	0.489	0.343
$\tau_{111,100}$	0.185	0.285	0.097	0.266	0.273	0.281	0.23	0.192	0.231	0.251 ‡	0.240	0.247 ‡
$\tau_{100,010}$	0.022	0.201	0.113	0.211	0.138	0.167	0.13	0.161	0.038	0.207 ‡	0.039	0.207 ‡
$\tau_{010,110}$	0.576	0.178	0.629	0.185	0.691	0.225	0.69	0.226	0.442	0.207	0.442	0.207
$-\log L$	3192.90		3192.57		3192.02		3191.55		3193.35		3193.3	

*: The resulting model is the same as the multinomial GLMM; $\pi_{1\cdot 1}$ ($\pi_{\cdot 11}$) and $\pi_{1\cdot 0}$ ($\pi_{\cdot 10}$) are the meta-analytic parameters of the TPF and FPF, respectively, for shortened humerous (shortened femur) of the fetus; π_{11d} is the meta-analytic parameter of the joint TPF ($d = 1$) or joint FPF ($d = 0$); Cln$\{\omega_1^\circ, \omega_2^\circ\} = \begin{cases} \text{Clayton rotated by } \omega_1^\circ & \text{if} \quad \tau > 0 \\ \text{Clayton rotated by } \omega_2^\circ & \text{if} \quad \tau < 0 \end{cases}$; ‡: We have substituted the BVN copula that interpolates from the Fréchet lower (perfect negative dependence) to the Fréchet upper (perfect positive dependence) bound as the Kendall's τ parameters were close to independence.

Supplementary Table 6: Maximized log-likelihoods, estimates and standard errors (SE) of the multinomial 1-truncated D-vine copula mixed models with beta margins for the accuracy of shortened humerus and shortened femur of the fetus in detecting Down syndrome in liveborn infants.

	BVN		Frank		$\text{Cln}\{0°, 90°\}$		$\text{Cln}\{0°, 270°\}$		$\text{Cln}\{180°, 90°\}$		$\text{Cln}\{180°, 270°\}$	
	Est.	SE	Est.	SE	Est.	SE	Est.	SE	Est.	SE	Est.	SE
π_{101}	0.091	0.044	0.088	0.042	0.091	0.044	0.092	0.044	0.089	0.043	0.090	0.043
π_{011}	0.086	0.019	0.087	0.019	0.086	0.019	0.086	0.019	0.086	0.019	0.086	0.019
π_{111}	0.292	0.045	0.294	0.044	0.299	0.043	0.299	0.043	0.295	0.043	0.295	0.043
π_{100}	0.024	0.006	0.023	0.006	0.024	0.006	0.024	0.006	0.024	0.006	0.024	0.006
π_{010}	0.054	0.008	0.050	0.006	0.051	0.007	0.051	0.007	0.053	0.008	0.053	0.008
π_{110}	0.034	0.006	0.033	0.005	0.034	0.006	0.034	0.006	0.033	0.005	0.033	0.005
$\pi_{1\cdot 1}$	0.383	0.045	0.382	0.044	0.391	0.047	0.391	0.047	0.385	0.043	0.385	0.043
$\pi_{\cdot 11}$	0.378	0.054	0.381	0.053	0.385	0.050	0.385	0.050	0.382	0.053	0.381	0.053
$\pi_{1\cdot 0}$	0.058	0.008	0.056	0.008	0.058	0.009	0.058	0.009	0.057	0.008	0.057	0.008
$\pi_{\cdot 10}$	0.088	0.012	0.083	0.010	0.085	0.011	0.085	0.011	0.086	0.011	0.086	0.011
γ_{101}	0.186	0.120	0.180	0.115	0.181	0.116	0.182	0.117	0.178	0.112	0.179	0.114
γ_{011}	0.016	0.020	0.016	0.020	0.017	0.020	0.016	0.020	0.016	0.020	0.015	0.019
γ_{111}	0.066	0.039	0.068	0.038	0.052	0.034	0.052	0.034	0.064	0.036	0.063	0.036
γ_{100}	0.015	0.008	0.015	0.008	0.015	0.008	0.015	0.008	0.015	0.008	0.015	0.008
γ_{010}	0.011	0.006	0.008	0.003	0.008	0.003	0.008	0.003	0.012	0.005	0.012	0.005
γ_{110}	0.010	0.006	0.009	0.005	0.010	0.006	0.010	0.006	0.009	0.005	0.009	0.005
$\tau_{101,011}$	-0.95	-	-0.95	-	-0.95	-	-0.95	-	-0.95	-	-0.95	-
$\tau_{011,111}$	0.300	0.324	0.310	0.307	0.178	0.307 ‡	0.178	0.305 ‡	0.371	0.270	0.371	0.273
$\tau_{111,100}$	0.197	0.275	0.128	0.297	0.304	0.228	0.304	0.225	0.221	0.255 ‡	0.220	0.254 ‡
$\tau_{100,010}$	-0.029	0.219	0.051	0.229	0.105	0.179	0.105	0.179	-0.026	0.238 ‡	-0.026	0.238 ‡
$\tau_{010,110}$	0.544	0.199	0.607	0.196	0.624	0.256	0.624	0.256	0.398	0.262	0.398	0.262
$-\log L$	3191.80		3191.31		3190.43		3190.44		3192.35		3192.36	

$\pi_{1\cdot 1}$ ($\pi_{\cdot 11}$) and $\pi_{1\cdot 0}$ ($\pi_{\cdot 10}$) are the meta-analytic parameters of the TPF and FPF, respectively, for shortened humerous (shortened femur) of the fetus; π_{11d} is the meta-analytic parameter of the joint TPF ($d = 1$) or joint FPF ($d = 0$); $\text{Cln}\{\omega_1^\circ, \omega_2^\circ\} = \begin{cases} \text{Clayton rotated by } \omega_1^\circ & \text{if} \quad \tau > 0 \\ \text{Clayton rotated by } \omega_2^\circ & \text{if} \quad \tau < 0 \end{cases}$; ‡: We have substituted the BVN copula that interpolates from the Fréchet lower (perfect negative dependence) to the Fréchet upper (perfect positive dependence) bound as the Kendall's τ parameters were close to independence.

Supplementary Table 7: Maximized log-likelihoods, estimates and standard errors in parentheses of bivariate copula mixed models for the accuracy of shortened humerus and shortened femur of the fetus in detecting Down syndrome in liveborn infants.

Copula	margin	log-likelihood	$\hat{\pi}_{1\cdot 1}$	$\hat{\pi}_{\cdot 11}$	$\hat{\pi}_{1\cdot 0}$	$\hat{\pi}_{\cdot 10}$
BVN*	normal	-159.57	0.367	0.357	0.049	0.074
			(0.039)	(0.054)	(0.009)	(0.011)
Frank		-159.70	0.356	0.343	0.047	0.071
			(0.040)	(0.049)	(0.008)	(0.010)
Clayton		-157.68	0.368	0.358	0.050	0.075
			(0.037)	(0.047)	(0.008)	0.010
Clayton by 180°		-161.97	0.361	0.351	0.048	0.072
			(0.040)	(0.055)	(0.009)	(0.012)
BVN	beta	-159.81	0.371	0.370	0.056	0.082
			(0.039)	(0.049)	(0.009)	(0.012)
Frank		-159.72	0.363	0.357	0.053	0.078
			(0.038)	(0.048)	(0.008)	(0.011)
Clayton		-157.56	0.375	0.375	0.056	0.083
			(0.037)	(0.044)	(0.008)	(0.010)
Clayton by 180°		-162.23	0.367	0.365	0.055	0.080
			(0.039)	(0.049)	(0.009)	(0.010)

*: The resulting model is the same as the bivariate GLMM; $\pi_{1\cdot 1}$ ($\pi_{\cdot 11}$) and $\pi_{1\cdot 0}$ ($\pi_{\cdot 10}$) are the meta-analytic parameters of the TPF and FPF, respectively, for shortened humerous (shortened femur) of the fetus.

References

Benacerraf, B., Nadel, A., and Bromley, B. (1994). Identification of second-trimester fetuses with autosomal trisomy by use of a sonographic scoring index. *Radiology*, 193(1):135–140.

Benacerraf, B., Neuberg, D., Bromley, B., and Frigoletto, J. F. (1992). Sonographic scoring index for prenatal detection of chromosomal abnormalities. *Journal of Ultrasound in Medicine*, 11(9):449–458.

Benacerraf, B., Neuberg, D., and Frigoletto, F.D., J. (1991). Humeral shortening in second-trimester fetuses with down syndrome. *Obstetrics and Gynecology*, 77(2):223–227.

Biagiotti, R., Periti, E., and Cariati, E. (1994). Humerus and femur length in fetuses with down syndrome. *Prenatal Diagnosis*, 14(6):429–434.

Bromley, B., Lieberman, E., and Benacerraf, B. (1997). The incorporation of maternal age into the sonographic scoring index for the detection at 14-20 weeks of fetuses with down's syndrome. *Ultrasound in Obstetrics and Gynecology*, 10(5):321–324.

Johnson, M., Michaelson, J., Barr, M., J., Treadwell, M., Hume, R.F., J., Dombrowski, M., and Evans, M. (1995). Combining humerus and femur length for improved ultrasonographic identification of pregnancies at increased risk for trisomy 21. *American Journal of Obstetrics and Gynecology*, 172(4):1229–1235.

Lockwood, C., Lynch, L., Ghidini, A., Lapinski, R., Berkowitz, G., Thayer, B., and Miller, W. (1993). The effect of fetal gender on the prediction of down syndrome by means of maternal serum α-fetoprotein and ultrasonographic parameters. *American Journal of Obstetrics and Gynecology*, 169(5):1190–1197.

Nyberg, D., Luthy, D., Resta, R., Nyberg, B., and Williams, M. (1998). Age-adjusted ultrasound risk assessment for fetal down's syndrome during the second trimester: Description of the method and analysis of 142 cases. *Ultrasound in Obstetrics and Gynecology*, 12(1):8–14.

Nyberg, D., Resta, R., Luthy, D., Hickok, D., and Williams, M. (1993). Humerus and femur length shortening in the detection of down's syndrome. *American Journal of Obstetrics and Gynecology*, 168(2):534–538.

Rodis, J., Vintzileos, A., Fleming, A., Ciarleglio, L., Nardi, D., Feeney, L., Scorza, W., Campbell, W., and Ingardia, C. (1991). Comparison of humerus length with femur length in fetuses with down syndrome. *American Journal of Obstetrics and Gynecology*, 165(4 PART 1):1051–1056.

Vintzileos, A., Egan, J., Smulian, J., Campbell, W., Guzman, E., and Rodis, J. (1996). Adjusting the risk for trisomy 21 by a simple ultrasound method using fetal long-bone biometry. *Obstetrics and Gynecology*, 87(6):953–958.
